# Supplementary material for: Health services satisfaction and medical exclusion among migrant youths in Gauteng Province of South Africa: A cross-sectional analysis of the GCRO survey (2017−2018)
Source: PLoS One. 2023 Nov 29;18(11):e0293958. doi: 10.1371/journal.pone.0293958 (PMC10686501; doi:10.1371/journal.pone.0293958)
Supplement: S1 File — (PDF) [file pone.0293958.s001.pdf]

Do-files

```
clear
use "gcro-qols-v-v1.1\gcro-qols-v-v1.1\qols-v-2017-2018-v1.1.dta"



*Survey weight
svyset, clear
svyset resp_id [pw=weight] , singleunit(certainty) strata(ward)

numlabel, add

*Wight [iw=weight]

ta Q15_02_age_recode
gen Agee=.
replace Agee=1 if Q15_02_age_recode==1
replace Agee=2 if Q15_02_age_recode==2
replace Agee=3 if Q15_02_age_recode==3
ta Agee

ta Q3_01_recode
recode Q3_01_recode (1=1 "Non-migrants") (2=2 "In-migrants") (3=3 "Immigrants"), gen (Migra_status)
ta Migra_status
ta Migra_status Q3_12_4

*Migrants not living with parents
ta Q3_12_4
gen MigraLess30=Migra_status if Agee==1 | Agee==2 | Agee==3
ta MigraLess30
ta MigraLess30 [iw=weight]
*Migrants below age 30 not living with parents, are the heads of households,their own primary caregivers, and use public, private and healthcare facilities from both public and private.
ta Q15_05_head
```

ta Q15_08_primary_carer

ta Q14_01_healthcare_services

gen migraYouthHH=MigraLess30 if Q15_05_head==1 | Q15_05_head==2 & Q15_08_primary_carer==1 & Q14_01_healthcare_services==1 | Q14_01_healthcare_services==2 | Q14_01_healthcare_services==3

ta migraYouthHH [iw=weight]

*GENERATE

gen MigraNon = migraYouthHH

ta MigraNon [iw=weight]

*Population group

ta A1_Pop_group

replace A1_Pop_group=. if A1_Pop_group==5

recode A1_Pop_group (1=1 "Black") (2/4=2 "Non_B"), gen (Pop_grou)

ta Pop_grou

recode Pop_grou (1/2=1 "filter"), gen (PopFilter)

ta PopFilter

*Education

ta Q15_01_education_recode

replace Q15_01_education_recode=. if Q15_01_education_recode==6

recode Q15_01_education_recode (1/3=1 "No_Primary") (4=2 "Secondary") (5=3 "Higher"), gen (Edicat)

ta Edicat

recode Edicat (1/3=1 "EduFilter"), gen (EduFilter)

ta EduFilter

*Medical Aid status

ta Q14_04_medaid

replace Q14_04_medaid=. if Q14_04_medaid==5 | Q14_04_medaid==6

ta Q14_04_medaid

recode Q14_04_medaid (1/3=1 "Yes") (4=2 "No"), gen (Med_Aid)

ta Med_Aid, nol

recode Med_Aid (1/2=1 "MediFilter"), gen (MediFilter)

ta MediFilter

*Health Status

recode Q14_11_health (1=1 "Excellent") (2=2 "Good") (3/4=3 "Poor"), gen (HealthStatus)

```
ta HealthStatus

*Disability Status

recode Q15_04_disability (1=1 "Not_disabled") (2/8=2 "Disabled"), gen (Disabiltty)

*Mental Health problem

recode Q14_16_mental_problems (0=1 "No") (1=2 "Yes"), gen (Mental_helth)

*Employment Status

recode Q11_12_working (0=1 "No") (1=2 "Yes"), gen (Working)

*Any income

replace Q15_20_income=. if Q15_20_income==18

recode Q15_20_income (17=1 "No_Income") (1/16=2 "Yes"), gen (IncomePerMonth)

*Satisfaction with health services

ta Q14_03_health_services

recode Q14_03_health_services (1/2=1 "satisfied") (3=2 "Neither") (4/5=3 "dissatisfied"), gen (HealthServSatisfaction)

ta HealthServSatisfaction

*Health care facility used

ta Q14_01_healthcare_services

replace Q14_01_healthcare_services=. if Q14_01_healthcare_services==4

replace Q14_01_healthcare_services=. if Q14_01_healthcare_services==5

replace Q14_01_healthcare_services=. if Q14_01_healthcare_services==6


*Outcome

ta Q14_05_access

ta Q9_11_alienation

ta Q14_06_reason_health_behavior

ta Q14_02_nonuse_public_health

gen MedicalEx=.

replace MedicalEx=0 if Q14_05_access==0 | Q14_05_access!=1

replace MedicalEx=0 if Q9_11_alienation==4 & Q14_05_access!=1

replace MedicalEx=0 if Q9_11_alienation==5 & Q14_05_access!=1

replace MedicalEx=1 if Q14_05_access==1 & Q14_05_access!=0

replace MedicalEx=1 if Q9_11_alienation==1 & Q14_05_access!=0

replace MedicalEx=1 if Q9_11_alienation==2 & Q14_05_access!=0

replace MedicalEx=1 if Q14_06_reason_health_behavior==7 & Q14_05_access!=0
```

```
replace MedicalEx=1 if Q14_06_reason_health_behavior==9 & Q14_05_access!=0
replace MedicalEx=1 if Q14_02_nonuse_public_health==5 & Q14_05_access!=0
replace MedicalEx=1 if Q14_02_nonuse_public_health==6 & Q14_05_access!=0
ta MedicalEx
gen MedicalExclusion=MedicalEx if MigraNon==1 | MigraNon==2 | MigraNon==3
ta MedicalExclusion
gen Age=Agee if MedicalExclusion==0 | MedicalExclusion==1
gen Sex=A2_Sex if MedicalExclusion==0 | MedicalExclusion==1
gen Population=Pop_grou if MedicalExclusion==0 | MedicalExclusion==1
ta Q3_13_Language
recode Q3_13_Language (5=1 "Zulu") (7=2 "Sesotho") (6=3 "Sepedi") (1/4=4 "Other") (8/13=4), gen (Language)
gen DominantLanguage=Language if MedicalExclusion==0 | MedicalExclusion==1
gen Education=Edicat if MedicalExclusion==0 | MedicalExclusion==1
gen HealthFacility=Q14_01_healthcare_services if MedicalExclusion==0 | MedicalExclusion==1
gen MedicalAid=Med_Aid if MedicalExclusion==0 | MedicalExclusion==1
gen HelthPst4=HealthStatus if MedicalExclusion==0 | MedicalExclusion==1
ta Q14_10_HIV_test
gen HIVTest12Months=Q14_10_HIV_test if MedicalExclusion==0 | MedicalExclusion==1
ta HIVTest12Months
gen HealthServices=HealthServSatisfaction if MedicalExclusion==0 | MedicalExclusion==1
ta Q14_09_9
recode Q14_09_9 (0=1) (1=2), gen (HIVStatus)
gen HIVStat=HIVStatus if MedicalExclusion==0 | MedicalExclusion==1
ta HIVStat
gen Disability=Disabiltty if MedicalExclusion==0 | MedicalExclusion==1
gen MentalPro=Mental_helth if MedicalExclusion==0 | MedicalExclusion==1
gen HealthServiceSati=HealthServSati if MedicalExclusion==0 | MedicalExclusion==1
gen WorkedPast7=Working if MedicalExclusion==0 | MedicalExclusion==1
gen Anyincome=.
replace Anyincome=1 if Q15_14_formal==0
replace Anyincome=1 if Q15_15_informal==0
replace Anyincome=1 if Q15_16_remittances==0
replace Anyincome=1 if Q15_17_support==0
```

```
replace Anyincome=1 if Q15_18_renting==0
replace Anyincome=1 if Q15_19_savings==0
replace Anyincome=2 if Q15_14_formal==1
replace Anyincome=2 if Q15_15_informal==1
replace Anyincome=2 if Q15_16_remittances==1
replace Anyincome=2 if Q15_17_support==1
replace Anyincome=2 if Q15_18_renting==1
replace Anyincome=2 if Q15_19_savings==1
ta Anyincome
gen Income=Anyincome if MedicalExclusion==0 | MedicalExclusion==1

*Dropping missing values
drop if MedicalExclusion==.
drop if  Age==.
drop if  Sex==.
drop if  Population==.
drop if DominantLanguage==.
drop if  Education==.
drop if HealthFacility==.
drop if  MedicalAid==.
drop if  HelthPst4==.
drop if  HIVTest12Months==.
drop if  HIVStat==.
drop if  Disability==.
drop if  MentalPro==.
drop if HealthServiceSati==.
drop if  WorkedPast==.
drop if  Income==.


*******************************************************************************************
****************************************************************

*Non-Migrants
gen GMedicalExclusion=MedicalExclusion if MigraNon==1
```

gen GAge=Age if MigraNon==1

gen GSex=Sex if MigraNon==1

gen GPopulation=Population if MigraNon==1

gen GDominantLanguage=DominantLanguage if MigraNon==1

gen GEducation=Education if MigraNon==1

gen GHealthFacility=HealthFacility if MigraNon==1

gen GMedicalAid=MedicalAid if MigraNon==1

gen GHelthPst4=HelthPst4 if MigraNon==1

gen GHIVTest12Months=HIVTest12Months if MigraNon==1

gen GHIVStat=HIVStat if MigraNon==1

gen GDisability=Disability if MigraNon==1

gen GMentalPro=MentalPro if MigraNon==1

gen GHealthServiceSati=HealthServiceSati if MigraNon==1

gen GWorkedPast=WorkedPast if MigraNon==1

gen GIncome=Income if MigraNon==1

*Summary statistics

sum GMedicalExclusion GAge GSex GPopulation GDominantLanguage GEducation GHealthFacility GMedicalAid GHelthPst4 GHIVTest12Months GHIVStat GDisability GMentalPro GHealthServiceSati GWorkedPast GIncome [aw=weight]

ci mean GMedicalExclusion GAge GSex GPopulation GDominantLanguage GEducation GHealthFacility GMedicalAid GHelthPst4 GHIVTest12Months GHIVStat GDisability GMentalPro GHealthServiceSati GWorkedPast GIncome [aw=weight]

*Univariate

ta GMedicalExclusion [iw=weight]

ta GAge [iw=weight]

ta GSex [iw=weight]

ta GPopulation [iw=weight]

ta GDominantLanguage [iw=weight]

ta GEducation [iw=weight]

ta GHealthFacility [iw=weight]

ta GMedicalAid [iw=weight]

ta GHelthPst4 [iw=weight]

ta GHIVTest12Months [iw=weight]

```
ta GHIVStat [iw=weight]
ta GDisability [iw=weight]
ta GMentalPro [iw=weight]
ta GHealthServiceSati [iw=weight]
ta GWorkedPast [iw=weight]
ta GIncome [iw=weight]

*Bi-variate
table Age MedicalExclusion MigraNon [iw=weight], row
table Sex MedicalExclusion MigraNon [iw=weight], row
table Population MedicalExclusion MigraNon [iw=weight], row
table DominantLanguage MedicalExclusion MigraNon [iw=weight], row
table Education MedicalExclusion MigraNon [iw=weight], row
table HealthFacility MedicalExclusion MigraNon [iw=weight], row
table MedicalAid MedicalExclusion MigraNon [iw=weight], row
table HelthPst4 MedicalExclusion MigraNon [iw=weight], row
table HIVTest12Months MedicalExclusion MigraNon [iw=weight], row
table HIVStat MedicalExclusion MigraNon [iw=weight], row
table Disability MedicalExclusion MigraNon [iw=weight], row
table MentalPro MedicalExclusion MigraNon [iw=weight], row
table HealthServiceSati MedicalExclusion MigraNon [iw=weight], row
table WorkedPast MedicalExclusion MigraNon [iw=weight], row
table Income MedicalExclusion MigraNon [iw=weight], row


*Internal migrants
gen IMedicalExclusion=MedicalExclusion if MigraNon==2
gen IAge=Age if MigraNon==2
gen ISex=Sex if MigraNon==2
gen IPopulation=Population if MigraNon==2
gen IDominantLanguage=DominantLanguage if MigraNon==2
gen IEducation=Education if MigraNon==2
gen IHealthFacility=HealthFacility if MigraNon==2
```

gen IMedicalAid=MedicalAid if MigraNon==2

gen IHelthPst4=HelthPst4 if MigraNon==2

gen IHIVTest12Months=HIVTest12Months if MigraNon==2

gen IHIVStat=HIVStat if MigraNon==2

gen IDisability=Disability if MigraNon==2

gen IMentalPro=MentalPro if MigraNon==2

gen IHealthServiceSati=HealthServiceSati if MigraNon==2

gen IWorkedPast=WorkedPast if MigraNon==2

gen IIncome=Income if MigraNon==2

*Summary statistics

sum IMedicalExclusion IAge ISex IPopulation IDominantLanguage IEducation IHealthFacility IMedicalAid IHelthPst4 IHIVTest12Months IHIVStat IDisability IMentalPro IHealthServiceSati IWorkedPast IIncome [aw=weight]

ci mean IMedicalExclusion IAge ISex IPopulation IDominantLanguage IEducation IHealthFacility IMedicalAid IHelthPst4 IHIVTest12Months IHIVStat IDisability IMentalPro IHealthServiceSati IWorkedPast IIncome [aw=weight]

*Univariate

ta IMedicalExclusion [iw=weight]

ta IAge [iw=weight]

ta ISex [iw=weight]

ta IPopulation [iw=weight]

ta IDominantLanguage [iw=weight]

ta IEducation [iw=weight]

ta IHealthFacility [iw=weight]

ta IMedicalAid [iw=weight]

ta IHelthPst4 [iw=weight]

ta IHIVTest12Months [iw=weight]

ta IHIVStat [iw=weight]

ta IDisability [iw=weight]

ta IMentalPro [iw=weight]

ta IHealthServiceSati [iw=weight]

ta IWorkedPast [iw=weight]

```
ta IIncome [iw=weight]

*Pearson chi2
tabulate IAge IMedicalExclusion, chi2
tabulate ISex IMedicalExclusion, chi2
tabulate IPopulation IMedicalExclusion, chi2
tabulate IDominantLanguage IMedicalExclusion, chi2
tabulate IEducation IMedicalExclusion, chi2
tabulate IHealthFacility IMedicalExclusion, chi2
tabulate IMedicalAid IMedicalExclusion, chi2
tabulate IHelthPst4 IMedicalExclusion, chi2
tabulate IHIVTest12Months IMedicalExclusion, chi2
tabulate IHIVStat IMedicalExclusion, chi2
tabulate IDisability IMedicalExclusion, chi2
tabulate IMentalPro IMedicalExclusion, chi2
tabulate IHealthServiceSati IMedicalExclusion, chi2
tabulate IWorkedPast IMedicalExclusion, chi2
tabulate IIncome IMedicalExclusion, chi2


*Immigrants
gen IIMedicalExclusion=MedicalExclusion if MigraNon==3
gen IIAge=Age if MigraNon==3
gen IISex=Sex if MigraNon==3
gen IIPopulation=Population if MigraNon==3
gen IIDominantLanguage=DominantLanguage if MigraNon==3
gen IIEducation=Education if MigraNon==3
gen IIHealthFacility=HealthFacility if MigraNon==3
gen IIMedicalAid=MedicalAid  if MigraNon==3
gen IIHelthPst4=HelthPst4 if MigraNon==3
gen IIHIVTest12Months=HIVTest12Months if MigraNon==3
gen IIHIVStat=HIVStat if MigraNon==3
gen IIDisability=Disability if MigraNon==3
```

gen IIMentalPro=MentalPro if MigraNon==3

gen IIHealthServiceSati=HealthServiceSati if MigraNon==3

gen IIWorkedPast=WorkedPast if MigraNon==3

gen IIIncome=Income if MigraNon==3

*Summary statistics

sum IIMedicalExclusion IIAge IISex IIPopulation IIDominantLanguage IIEducation IIHealthFacility IIMedicalAid IIHelthPst4 IIHIVTest12Months IIHIVStat IIDisability IIMentalPro IIHealthServiceSati IIWorkedPast IIIncome [iw=weight]

ci means IIMedicalExclusion IIAge IISex IIPopulation IIDominantLanguage IIEducation IIHealthFacility IIMedicalAid IIHelthPst4 IIHIVTest12Months IIHIVStat IIDisability IIMentalPro IIHealthServiceSati IIWorkedPast IIIncome [aw=weight]

*Univariate

ta IIMedicalExclusion [iw=weight]

ta IIAge [iw=weight]

ta IISex [iw=weight]

ta IIPopulation [iw=weight]

ta IIDominantLanguage [iw=weight]

ta IIEducation [iw=weight]

ta IIHealthFacility [iw=weight]

ta IIMedicalAid [iw=weight]

ta IIHelthPst4 [iw=weight]

ta IIHIVTest12Months [iw=weight]

ta IIHIVStat [iw=weight]

ta IIDisability [iw=weight]

ta IIMentalPro [iw=weight]

ta IIHealthServiceSati [iw=weight]

ta IIWorkedPast [iw=weight]

ta IIIncome [iw=weight]

*Pearson chi2

tabulate IIAge IIMedicalExclusion, chi2

tabulate IISex IIMedicalExclusion, chi2

tabulate IIPopulation IIMedicalExclusion, chi2

```
tabulate IIDominantLanguage IIMedicalExclusion, chi2
tabulate IIEducation IIMedicalExclusion, chi2
tabulate IIHealthFacility IIMedicalExclusion, chi2
tabulate IIMedicalAid IIMedicalExclusion, chi2
tabulate IIHelthPst4 IIMedicalExclusion, chi2
tabulate IIHIVTest12Months IIMedicalExclusion, chi2
tabulate IIHIVStat IIMedicalExclusion, chi2
tabulate IIDisability IIMedicalExclusion, chi2
tabulate IIMentalPro IIMedicalExclusion, chi2
tabulate IIHealthServiceSati IIMedicalExclusion, chi2
tabulate IIWorkedPast IIMedicalExclusion, chi2
tabulate IIIncome IIMedicalExclusion, chi2


....................................................
*In-migrants and Immigrants as Migrants
gen MMedicalExclusion=MedicalExclusion if MigraNon==2 | MigraNon==3
gen MAge=Age if MigraNon==2 | MigraNon==3
gen MSex=Sex if MigraNon==2 | MigraNon==3
gen MPopulation=Population if MigraNon==2 | MigraNon==3
gen MDominantLanguage=DominantLanguage if MigraNon==2 | MigraNon==3
gen MEducation=Education if MigraNon==2 | MigraNon==3
gen MHealthFacility=HealthFacility if MigraNon==2 | MigraNon==3
gen MMedicalAid=MedicalAid  if MigraNon==2 | MigraNon==3
gen MHelthPst4=HelthPst4 if MigraNon==2 | MigraNon==3
gen MHIVTest12Months=HIVTest12Months if MigraNon==2 | MigraNon==3
gen MHIVStat=HIVStat if MigraNon==2 | MigraNon==3
gen MDisability=Disability if MigraNon==2 | MigraNon==3
gen MMentalPro=MentalPro if MigraNon==2 | MigraNon==3
gen MHealthServiceSati=HealthServiceSati if MigraNon==2 | MigraNon==3
gen MWorkedPast=WorkedPast if MigraNon==2 | MigraNon==3
gen MIncome=Income if MigraNon==2 | MigraNon==3
gen Migra=.
replace Migra=1 if MigraNon==2
```

replace Migra=2 if MigraNon==3

ta Migra [iw=weight]

recode Migra (1=1 "In-migrant") (2=2 "Immigrant"), gen (Migration_Status)

*Summary statistics

sum Migration_Status MMedicalExclusion MAge MSex MPopulation MDominantLanguage MEducation MHealthFacility MMedicalAid MHelthPst4 MHIVTest12Months MHIVStat MDisability MMentalPro MHealthServiceSati MWorkedPast MIncome [aw=weight]

ci mean Migration_Status MMedicalExclusion MAge MSex MPopulation MDominantLanguage MEducation MHealthFacility MMedicalAid MHelthPst4 MHIVTest12Months MHIVStat MDisability MMentalPro MHealthServiceSati MWorkedPast MIncome [aw=weight]

*Univariate

ta Migration_Status [iw=weight]

ta MMedicalExclusion [iw=weight]

ta MAge [iw=weight]

ta MSex [iw=weight]

ta MPopulation [iw=weight]

ta MDominantLanguage [iw=weight]

ta MEducation [iw=weight]

ta MHealthFacility [iw=weight]

ta MMedicalAid [iw=weight]

ta MHelthPst4 [iw=weight]

ta MHIVTest12Months [iw=weight]

ta MHIVStat [iw=weight]

ta MDisability [iw=weight]

ta MMentalPro [iw=weight]

ta MHealthServiceSati [iw=weight]

ta MWorkedPast [iw=weight]

ta MIncome [iw=weight]

*Pearson chi2

tabulate MAge MMedicalExclusion, chi2

tabulate MSex MMedicalExclusion, chi2

tabulate MPopulation MMedicalExclusion, chi2

tabulate MDominantLanguage MMedicalExclusion, chi2

tabulate MEducation MMedicalExclusion, chi2

tabulate MHealthFacility MMedicalExclusion, chi2

tabulate MMedicalAid MMedicalExclusion, chi2

tabulate MHelthPst4 MMedicalExclusion, chi2

tabulate MHIVTest12Months MMedicalExclusion, chi2

tabulate MHIVStat MMedicalExclusion, chi2

tabulate MDisability MMedicalExclusion, chi2

tabulate MMentalPro MMedicalExclusion, chi2

tabulate MHealthServiceSati MMedicalExclusion, chi2

tabulate MWorkedPast MMedicalExclusion, chi2

tabulate MIncome MMedicalExclusion, chi2

tabulate Migration_Status MMedicalExclusion, chi2

*Unadjusted

svy: logit MMedicalExclusion i.MAge, or

svy: logit MMedicalExclusion i.MSex, or

svy: logit MMedicalExclusion i.MPopulation, or

svy: logit MMedicalExclusion i.MDominantLanguage, or

svy: logit MMedicalExclusion i.MEducation, or

svy: logit MMedicalExclusion i.MHealthFacility, or

svy: logit MMedicalExclusion i.MMedicalAid, or

svy: logit MMedicalExclusion i.MHelthPst4, or

svy: logit MMedicalExclusion i.MHIVTest12Months, or

svy: logit MMedicalExclusion i.MHIVStat, or

svy: logit MMedicalExclusion i.MDisability, or

svy: logit MMedicalExclusion i.MMentalPro, or

svy: logit MMedicalExclusion i.MHealthServiceSati, or

svy: logit MMedicalExclusion i.MWorkedPast, or

svy: logit MMedicalExclusion i.MIncom, or

svy: logit MMedicalExclusion i.Migration_Status, or

*Adjusted regression and vif

svy: logit MMedicalExclusion i.MAge i.MSex i.MPopulation i.MDominantLanguage i.MEducation i.MHealthFacility i.MMedicalAid i.MHelthPst4 i.MHIVTest12Months i.MHIVStat i.MDisability i.MMentalPro i.MHealthServiceSati i.MWorkedPast i.MIncome i.Migration_Status, or

regress MMedicalExclusion i.MAge i.MSex i.MPopulation i.MDominantLanguage i.MEducation i.MHealthFacility i.MMedicalAid i.MHelthPst4 i.MHIVTest12Months i.MHIVStat i.MDisability i.MMentalPro i.MHealthServiceSati i.MWorkedPast i.MIncome i.Migration_Status

vif
