# Supplementary material for: Health services satisfaction and medical exclusion among migrant youths in Gauteng Province of South Africa: A cross-sectional analysis of the GCRO survey (2017−2018)
Source: PLoS One. 2023 Nov 29;18(11):e0293958. doi: 10.1371/journal.pone.0293958 (PMC10686501; doi:10.1371/journal.pone.0293958)
Supplement: Attachment — Submitted filename: PONE-D-22-33773.pdf [file pone.0293958.s003.pdf]

PLOS ONE

Health Services Satisfaction and Medical Exclusion among Migrant Youths in Gauteng Province of South Africa: A Cross-sectional Study of the GCRO Survey (2017−2018)

--Manuscript Draft--

**Manuscript Number:**	PONE-D-22-33773
**Article Type:**	Research Article
**Full Title:**	Health Services Satisfaction and Medical Exclusion among Migrant Youths in Gauteng Province of South Africa: A Cross-sectional Study of the GCRO Survey (2017−2018)
**Short Title:**	Health Services Satisfaction and Medical Exclusion among Migrant Youths in Gauteng
**Corresponding Author:**	Monica Ewomazino Akokuwebe, Ph.D.<br>North-West University - Mafikeng Campus<br>Mafikeng, North West SOUTH AFRICA
**Keywords:**	Factors; health services; migrants; medical exclusion; South Africa; youth
**Abstract:**	Background<br>The phenomenon of medical exclusion and dissatisfaction of healthcare services is very real in the contemporary South African public health care system. Medical mistreatment of migrant youths can be attributed to “social phobia” acting as an obstacle to health services’ satisfaction. This study aimed to determine the prevalence and factors contributing to medical exclusion among migrant youths in Gauteng Province, South Africa.<br>Methods: The Round 5 Gauteng City-Region Observatory (GCRO) Quality of Life (QoL) survey was conducted in 2017–2018, a nationally representative survey piloted every two years in South Africa, was utilized in this study. A 2-year cohort study of 24,889 respondents aged 18 to 29 commenced in October 2017 up to September 2018, and baseline data consisted of 4872 respondents, comprising non-migrants, in-migrants and immigrants, from where 2162 in-migrants and immigrants were utilized as the sample size. The data was analysed using descriptive statistics, Chi-Square analysis and logistic regression.<br>Results: A total of 2162 migrants, comprising 35.4% in-migrants and 9.0% of immigrants, from the 4872 respondents, were included in the analysis. The prevalence of medical exclusion of in-migrant and immigrant youths were 5.5% and 4.2%, and the majority of them reported the use of public health facilities (in-migrants – 84.3% vs. immigrants – 87.1%). At the bivariate level, demographic (age, sex, and population group), economic (employed and any income) and health-related (no medical aid and household member with mental health) factors were significantly associated with medical exclusion (ρ≤0.05, ρ≤0.01 ρ≤0.001). The adjusted odds ratio (AOR) of logistic regression indicated factors such as female gender (AOR 1.07, 95% CI 0.678, 1.705), no medical aid cover (AOR 1.23, 95% CI 0.450–3.362), and neither (AOR 1.59, 95% CI 0.606–4.174) or dissatisfied (AOR 4.29, 95% CI 2.528–7.270) with the health services were predictors of medical exclusion.<br>Conclusion: Having no medical aid cover, being a female and dissatisfied, or neither satisfied nor dissatisfied with health services significantly increased the odds of medical exclusion among migrant youths. To increase healthcare utilization and ensuring adequate medical care of migrant youths, easy access to medical aid cover without increasing costs should be guaranteed. Therefore, there should be no consequences for lack of residence status or correct documentation papers when accessing healthcare services among migrant youths in South Africa.
**Order of Authors:**	Monica Ewomazino Akokuwebe, Ph.D.
	Salmon Likoko, M.Sc.
	Erhabor Sunday Idemudia, Ph.D.
	Godswill Nwabuisi Osuafor, Ph.D.
**Additional Information:**	
**Question**	**Response**

Powered by Editorial Manager® and ProduXion Manager® from Aries Systems Corporation

**Financial Disclosure**<br><br>Enter a financial disclosure statement that describes the sources of funding for the work included in this submission. Review the submission guidelines for detailed requirements. View published research articles from *PLOS ONE* for specific examples.<br><br>This statement is required for submission and **will appear in the published article** if the submission is accepted. Please make sure it is accurate.<br><br>**Unfunded studies**<br>Enter: *The author(s) received no specific funding for this work.*<br><br>**Funded studies**<br>Enter a statement with the following details:<br>• Initials of the authors who received each award<br>• Grant numbers awarded to each author<br>• The full name of each funder<br>• URL of each funder website<br>• Did the sponsors or funders play any role in the study design, data collection and analysis, decision to publish, or preparation of the manuscript?<br>• **NO** - Include this sentence at the end of your statement: *The funders had no role in study design, data collection and analysis, decision to publish, or preparation of the manuscript.*<br>• **YES** - Specify the role(s) played.<br><br>* typeset	The author(s) received no specific funding for this work.
**Competing Interests**<br><br>Use the instructions below to enter a competing interest statement for this submission. On behalf of all authors, disclose any competing interests that could be perceived to bias this work—acknowledging all financial support and any other relevant financial or non-financial competing interests.<br><br>This statement is required for submission	The authors have declared that no competing interests exist.

and **will appear in the published article** if the submission is accepted. Please make sure it is accurate and that any funding sources listed in your Funding Information later in the submission form are also declared in your Financial Disclosure statement.<br><br>View published research articles from *PLOS ONE* for specific examples.<br><br>**NO authors have competing interests**<br><br>Enter: *The authors have declared that no competing interests exist.*<br><br>**Authors with competing interests**<br><br>Enter competing interest details beginning with this statement:<br><br>*I have read the journal's policy and the authors of this manuscript have the following competing interests: [insert competing interests here]*<br><br>* typeset	
**Ethics Statement**<br><br>Enter an ethics statement for this submission. This statement is required if the study involved:<br><br>• Human participants<br>• Human specimens or tissue<br>• Vertebrate animals or cephalopods<br>• Vertebrate embryos or tissues<br>• Field research<br><br>Write "N/A" if the submission does not require an ethics statement.<br><br>General guidance is provided below. Consult the submission guidelines for detailed instructions. **Make sure that all information entered here is included in the Methods section of the manuscript.**	This study only makes use of secondary data without involving any human subjects. Therefore, no formal ethical approval was required. However, the permission to use the data was sought from the GCRO through a written request. Permission was given subject to using the data for this particular research topic only and publishing the findings in a peer-reviewed journal.

**Format for specific study types**<br><br>**Human Subject Research (involving human participants and/or tissue)**<br>• Give the name of the institutional review board or ethics committee that approved the study<br>• Include the approval number and/or a statement indicating approval of this research<br>• Indicate the form of consent obtained (written/oral) or the reason that consent was not obtained (e.g. the data were analyzed anonymously)<br><br>**Animal Research (involving vertebrate animals, embryos or tissues)**<br>• Provide the name of the Institutional Animal Care and Use Committee (IACUC) or other relevant ethics board that reviewed the study protocol, and indicate whether they approved this research or granted a formal waiver of ethical approval<br>• Include an approval number if one was obtained<br>• If the study involved *non-human primates*, add *additional details* about animal welfare and steps taken to ameliorate suffering<br>• If anesthesia, euthanasia, or any kind of animal sacrifice is part of the study, include briefly which substances and/or methods were applied<br><br>**Field Research**<br><br>Include the following details if this study involves the collection of plant, animal, or other materials from a natural setting:<br>• Field permit number<br>• Name of the institution or relevant body that granted permission	
**Data Availability**<br><br>Authors are required to make all data underlying the findings described fully available, without restriction, and from the time of publication. PLOS allows rare exceptions to address legal and ethical concerns. See the PLOS Data Policy and FAQ for detailed information.	Yes - all data are fully available without restriction

A Data Availability Statement describing where the data can be found is required at submission. Your answers to this question constitute the Data Availability Statement and **will be published in the article**, if accepted.<br><br>**Important:** Stating 'data available on request from the author' is not sufficient. If your data are only available upon request, select 'No' for the first question and explain your exceptional situation in the text box.<br><br>Do the authors confirm that all data underlying the findings described in their manuscript are fully available without restriction?	
**Describe where the data may be found in full sentences. If you are copying our sample text, replace any instances of XXX with the appropriate details.**<br><br>• If the data are **held or will be held in a public repository**, include URLs, accession numbers or DOIs. If this information will only be available after acceptance, indicate this by ticking the box below. For example: *All XXX files are available from the XXX database (accession number(s) XXX, XXX.).*<br>• If the data are all contained **within the manuscript and/or Supporting Information files**, enter the following: *All relevant data are within the manuscript and its Supporting Information files.*<br>• If neither of these applies but you are able to provide **details of access elsewhere**, with or without limitations, please do so. For example:<br><br>*Data cannot be shared publicly because of [XXX]. Data are available from the XXX Institutional Data Access / Ethics Committee (contact via XXX) for researchers who meet the criteria for access to confidential data.*<br><br>*The data underlying the results presented in the study are available from (include the name of the third party*	The data underlying the results presented in the study are available from (https://www.gcro.ac.za).

and contact information or URL).<br>• This text is appropriate if the data are owned by a third party and authors do not have permission to share the data.<br><br>* typeset	
Additional data availability information:	Tick here if your circumstances are not covered by the questions above and you need the journal's help to make your data available.

Powered by Editorial Manager® and ProduXion Manager® from Aries Systems Corporation

Manuscript

Click here to access/download;Manuscript;Edited 16th Paper.docx

Health Services Satisfaction and Medical Exclusion among Migrant Youths in Gauteng Province of South Africa: A Cross-sectional Study of the GCRO Survey (2017–2018)

Monica Ewomazino Akokuwebe[1]*, Salmon Likoko[2], Erhabor Sunday Idemudia[1] and Godswill Nwabuisi Osuafor[1]

[1]*Faculty of Humanities, North-West University, Mafikeng, South Africa*. [2] Statistics South Africa, ISIbalo House, Koch Street Salvokop, Pretoria. [3]*Population Studies and Demography, Faculty of Humanities, North-West University, Mafikeng, South Africa*

* Correspondence Email: nwupostdoctoral2021@gmail.com; Phone: +27840633819

Abstract

Background
The phenomenon of medical exclusion and dissatisfaction of healthcare services is very real in the contemporary South African public health care system. Medical mistreatment of migrant youths can be attributed to "social phobia" acting as an obstacle to health services' satisfaction. This study aimed to determine the prevalence and factors contributing to medical exclusion among migrant youths in Gauteng Province, South Africa.

Methods: The Round 5 Gauteng City-Region Observatory (GCRO) Quality of Life (QoL) survey was conducted in 2017–2018, a nationally representative survey piloted every two years in South Africa, was utilized in this study. A 2-year cohort study of 24,889 respondents aged 18 to 29 commenced in October 2017 up to September 2018, and baseline data consisted of 4872 respondents, comprising non-migrants, in-migrants and immigrants, from where 2162 in-migrants and immigrants were utilized as the sample size. The data was analysed using descriptive statistics, Chi-Square analysis and logistic regression.

Results: A total of 2162 migrants, comprising 35.4% in-migrants and 9.0% of immigrants, from the 4872 respondents, were included in the analysis. The prevalence of medical exclusion of in-migrant and immigrant youths were 5.5% and 4.2%, and the majority of them reported the use of public health facilities (in-migrants – 84.3% vs. immigrants – 87.1%). At the bivariate level, demographic (age, sex, and population group), economic (employed and any income) and health-related (no medical aid and household member with mental health) factors were significantly associated with medical exclusion ($\rho \leq 0.05$, $\rho \leq 0.01$ $\rho \leq 0.001$). The adjusted odds ratio (AOR) of logistic regression indicated factors such as female gender (AOR 1.07, 95% CI 0.678, 1.705), no medical aid cover (AOR 1.23, 95% CI 0.450–3.362), and neither (AOR 1.59, 95% CI 0.606–4.174) or dissatisfied (AOR 4.29, 95% CI 2.528–7.270) with the health services were predictors of medical exclusion.

Conclusion: Having no medical aid cover, being a female and dissatisfied, or neither satisfied nor dissatisfied with health services significantly increased the odds of medical exclusion among migrant youths. To increase healthcare utilization and ensuring adequate medical care of migrant youths, easy access to medical aid cover without increasing costs should be guaranteed. Therefore, there should be no consequences for lack of residence status or correct documentation papers when accessing healthcare services among migrant youths in South Africa.

Introduction

Across the globe, including the African continent, migration is being influenced significantly by economic instability, poverty, armed conflict and unrest, as well as social, political, and technological transformations [1-3]. Critical conditions push people from their home countries to seek a better life elsewhere. As a result of this mass relocation of people, public resources will be challenged in the different countries to which they are relocating. This is both during their movement and upon their arrival. Regardless of their gender, they are often victims of violence, infectious diseases, and malnutrition, which frequently accompany displaced populations and migrant movements [4,5]. Migrants nevertheless face more risks to their safety and wellbeing than the ordinary non-migrant, due to the abrupt and dramatic onset of emergencies and related uncertainty connected with migrant status, despite the massive efforts made to meet their health requirements [6,7]. However, the complex interactions between the status of migrants and their health indicate that these may have either positive or negative effects on their overall well-being [8-11]. Therefore, it is critical for migrants to have access to healthcare services in their host countries.

The International Organization for Migration (2020) survey report showed that the present-day African migrants are largely youths, since 60.0% of irregular African migrants are under 35 years; and 27.5% of migrants aged 15–29 years are hosted in several African countries [1,5]. The trend and pattern of migration ranges from low-income to high-income nations, including those African countries with political, social and economic stability, envisages a growing prevalence of migrant youths. Nearly 39.5 million Africans have migrated, of which 21 million did so within the continent and 18.5 million outside of it [3,5]. In Africa, labour migration is largely intra-regional (80%), of which a majority are low-skilled labour migrants. In addition, of great importance in the region, is the alliance of South-South migration corridors to nearby markets in search of employment with better remuneration [11-13]. African migrants transiting to other countries are in high demand in economic sectors. These economic sectors are significant drivers for heavy flow of migration routes within or outside the African continent. Similarly, South Africa has been the destination for most migrants from other African countries, with agreement ties with the United Nations High Commissioner for Refugees (UNHCR) to manage migrant-related issues [14]. Even though their contributions to the economic, sociocultural, and political development of their host nations are seldom publicly acknowledged, migrant people nevertheless experience social marginalization and poor health [15]. These challenges are multifaceted, ranging from racial and ethnic to generational differences. Primarily, the poor health outcomes of migrants can be construed from how they juggle multiple menial jobs to earn the income required to meet their upkeep and contributions to their households [16-17]. Thus, it has been established that existing health policy responses do not extend to migrant patients, hence migrants face myriad problems when they try to access public health services in South Africa. But the right to have access to health care services is a basic fundamental human right guaranteed by the Constitution of any nation [18]. In South Africa, the Human Rights Commission states Section 27 of the Constitution that provides that: "*everyone has the right to have access to healthcare services, including reproductive health care services, and no one may be refused emergency medical treatment*" [19].

Factors associated with migrants' exclusion from healthcare access have been cited in several studies [20-22]. First, pre-migration factors (trauma experience) and post-migration challenges (access to healthcare services) have been reported to majorly contribute negatively to migrants' health [9,23]. Other factors that may cause medical exclusion of migrants include poor knowledge of migrant rights, low socio-economic status, cultural variety and interests, religious beliefs, language barriers, and poor understanding of the healthcare systems in the host countries of the migrants [24,25]. Other studies have mentioned several barriers hindering migrants from accessing healthcare, such as cost, health system

complexity, incompetent health workers, long queues and wait times, discriminatory attitudes or mannerisms of health workers towards migrants with chronic ailments (such as HIV, TB, diabetes etc.), and fear among undocumented immigrants [26,27]. Moreover, quite a number of existing studies have showed that socio-demographic factors such as educational level, income, employment, age, health insurance coverage, and surety (parent, guardian, or guarantor) have been mentioned as important social determinants of health [8]. Thus, socioeconomic determinants of health play a key role in the decline of migrants' health after a period in their host nations [25], and this is particularly true for African migrants [3].

Also, other studies have mentioned medical xenophobia as one of the major barriers that migrants are faced with and this has been a hindrance to healthcare accessibility in South Africa. Medical xenophobia has been labelled as having the forms of negative attitudes, unsupportive practices, denied access to any form of medical treatment or care, stigmatization, structural violence, stereotyping, and blaming migrants for their destitution [24,28]. Also, there is a misconception that foreign-born migrants have deprived South African nationals of employment and other business opportunities, as they felt that migrants are posing a strain on limited social services and amenities, and this has constituted the main drivers of xenophobia [26,27]. However, the South African government has taken steps to ratify employment laws that are not in favour of migrants. Structural and practical xenophobia scenarios have plunged immigrants into poverty and misery, preventing them from access to all social services. Studies have indicated that undocumented migrants, asylum-seekers and refugees come from diverse African countries that are plagued with endemic and chronic diseases [29,30], while the burden of non-communicable diseases is found among foreign-born migrants [31-33].

Despite the said reviewed studies, there is a gap in studies and methodological approach that seek to fill the gap that shows how migrant youths are prevented from using healthcare services. Thus, the objective of this study is to examine the predictors associated with health service satisfaction and medical exclusion among migrant youths in South Africa. Findings from this study will help to redesign the existing practical interventions that addresses the unmet health needs of youth migrants in South Africa.

Methods and Materials

Study area

The study area is the South African province called Gauteng, whose name means 'golden place' in Sotho-Tswana. South Africa is the southernmost country in Africa, with a population of over 60 million people and an area of 1,221,037 square kilometres [34]. Gauteng Province is bordered by the Free State, North West, Limpopo and Mpumalanga Provinces, and it is the smallest province, covering an area of 18 178km², approximately 1.4% of the total surface area of South Africa, and the most populous being home to 15.8 million people, with a demographically youthful population with a median age of 28 [35,36]. Geographically, Gauteng lies on the highest part of the interior plateau, on the rolling plains of South Africa’s Highveld; its capital is Johannesburg and it also contains the city of Pretoria, as well as the East Rand, West Rand, and Vaal areas [37]. Gauteng Province is divided into three metropolitan municipalities, namely: the City of Tshwane, the City of Ekurhuleni, and the City of Johannesburg, as well as two district municipalities: West Rand and Sedibeng, which are further subdivided into six local municipalities: Mogale City, Rand West City, Merafong, Emfuleni, Midvaal and Lesedi (Fig. 1) [38,39]. Gauteng Province is the powerhouse and economic hub of the country and sub-continent, responsible for over 34.8% of the gross domestic product (GDP) as well as being the heart of the commercial, business, and industrial sectors of South Africa. The most important industries, including real estate, business, finance, general government services, and manufacturing services, are located in

Gauteng, which is also the financial services centre of Africa. These industries all contribute to Gauteng's GDP [39,40].

To a great extent, the majority of the foreign banks have their head offices, in Gauteng Province, as well as a number of South African banks, stockbrokers, and insurance giants. However, gold mining constitutes about 80% of Gauteng's mineral production output and the biggest gold and diamond mining houses such as Anglo American and De Beers are situated in Johannesburg, the capital of Gauteng Province [40]. Furthermore, South Africa at present is plagued with persistent droughts and water scarcities which predominantly influence periodic labour migration. Migration within and outside countries in South Africa is driven largely by the pursuit of economic opportunities, political uncertainty and, increasingly, environmental hazards [41,42]. Thus, industrial developments such as the mining sectors in South Africa, Botswana and Zambia, and the oil wealth of Angola, have been an attractive feature for both skilled and unskilled labour migrants from within the region and elsewhere [14,38]. According to Statistics South Africa, a net inflow of 852,992 foreign nationals was predicted for the 2016–2020 period, a decrease from the 916,346 forecast for the 2011–2016 period. In addition, SA Statistics (2020) reported an estimate of 2.9 million migrants who are presently residing in South Africa at mid-year 2020 [7]. Five Provinces, out of the nine Provinces, have experienced a net influx of people moving, including both internal and international migrants. These are Gauteng, the Western Cape, the North West, Mpumalanga and the Northern Cape [39]. Gauteng has seen the greatest influx of persons since 2016, and more than three times the numbers seen in the Western Cape Province, which had the second higher number of migrants [40]. Also, persons from all provinces, including more than half of the international migrants, are moving to Gauteng, as a result of the economic strength of the Province and job opportunities prospects, which has made the province an attractive destination.

Study design and data source

The Round 5 Gauteng City-Region Observatory (GCRO) Quality of Life (QoL) survey was conducted in 2017–2018, a nationally representative survey piloted every two years in South Africa, was utilized in this study. The GCRO engaged a multi-stage and stratified-cluster sampling design, which was used in clustering sampling of households, by random selection of 529 wards utilized as primary sampling units. The Round 5 GCRO's QoL survey was conducted from October 2017 to September 2018 to collect data from the nine municipalities of Gauteng Province. The 2017–2018 GCRO QoL survey data is made available to principal investigators based on written request via the GCRO website (https://www.gcro.ac.za), subsequent to ethical concerns such as voluntary participation, informed consent, respect for persons, anonymity, confidentiality, potential for harm, and communication of results for communication. At the sampling level, the first stage was guided by the 2011 South Africa Population Housing Census (PHC) of the definition of enumeration areas (EAs), where EAs were identified and sampled within the selected primary sampling units (PSUs). In the second stage, the cataloguing of households was carried out in each EA sampled, and a sample of households were selected using systematic random sampling.

In addition, all members in each household who met the inclusion criterion (aged 18-29 years) were eligible to participate in the survey. The sample clusters were dispersed across the urban and rural strata within each municipality of the sampled EAs, proportionate to the size of the associated populations within the sampling frame. Clusters (primary sampling units) were assigned to the city and local municipalities within the strata in proportion to the number of households in the census frame for each stratum within the Province. Further, survey locations were selected from the dataset of residential dwellings of the 2011 National Census, where respondents were randomly selected by a data collection application called ResearchGo. A sampling frame of all residential structures in Gauteng was achieved using the Geo Terra Image (GTI) and Building-Based Land-Use (BBLU) layer. The Round 5 GCRO QoL 2017-2018 survey collected information from respondents regarding socio-demographic, health-

related, socio-economic circumstances, quality of life, attitudes to service delivery, psycho-social attitudes, value-base, and other characteristics. The final samples of the GCRO QoL survey 2017–2018 were 24,889 respondents collected from 52 sampled wards in municipalities of Gauteng Province of South Africa. However, the data analyzed in this study were limited to a total of 2,162 immigrants and in-migrants in order to eliminate any potential recall bias. For the 2017–2018 GCRO QoL study, a thorough report on the context, sampling design, questionnaires, sampling frame, data collection techniques, and ethical approval was previously published [44].

Study population and sample size

In this study, the population were migrants aged 18–29 years, stratified by in-migrants and immigrants. This study's conceptual clarification and operationalization showed that in-migrants are South African nationals that move within or from one Province to another in search of prospective economic opportunities, while immigrants are non-South African nationals who move across international borders for the purposes of economic pursuits and settlement. The total number of the migrant population aged 18–29 years in 2017–2018 was 2,162, made up of 1,725 in-migrants and 437 immigrants. In order to expand the range of generalization, both migrant groups were used as the target population to increase the level of precision, therefore, justifying its use for the study. Also, several studies conducted on migration reported a greater influx of in-migrants from rural to urban areas or from non-industrial to industrial areas in search of better job prospects, as this finding has administratively been documented in migration studies conducted in South Africa, hence their large population [3,26]. Similarly, the population size of immigrants is small as reported by quite a number of studies conducted in South Africa, although this report is not a true reflection of the immigrant population as they are vulnerable to discrimination and exploitation, since the majority of them are poor, illiterate, and reside in slums or shelters [4]. Ineffective policy legislation and administration are not meeting the needs and settlements of immigrants, leading to their poor documentation and insignificant population size for fear of being arrested and deported by the immigration officials in South Africa.

Variable measurements

Outcome variable

The outcome variable for this study was Medical Exclusion, defined as denial of access or treatment, discretionary health care access, using a derogatory name or words ('*makwerekwere*', a term used to refer to foreigners), negative attitudes and practices of health professionals and employees towards migrants based purely on their identity as non-South Africans [45,46]. The outcome variable medical exclusion was re-categorized as 'Yes' (medically excluded, coded as '1') and 'No' (not medically excluded, coded as '0') for use in logistic regression analyses. The 2017–2018 GCRO QoL survey included various questions, including one that asked if anyone in the household needed healthcare in the last year but was unable to obtain it [44]. This was generated from the responses of respondents such as: In the past 12 months, 'was there anybody in this household who needed healthcare but was unable to get it (Q14_05_access)', 'nobody cares about people like me (Q9_11_alienation)', 'what was the main reason that person was unable to get the health care they needed (Q14_06_reason_health_behaviour)', and 'what is the main reason that you don't use public health facilities (Q14_02_nonuse_ public_ health)'. Thus, the outcome variable is binary in nature and two response categories were created to indicate 'not medically excluded' (No = '0') and 'medically excluded' (Yes = '1'). So 'not medically excluded' category coded as '0' was derived from respondents' responses who said 'no', 'disagree', or 'strongly disagree'' to the question asked: 'In the past 12 months, was there anybody in this household who needed healthcare but was unable to get it?' and 'nobody cares about people like me'. The 'medically excluded' category coded as '1' was also derived from responses of respondents who said 'yes', 'agree', 'strongly agree' 'nobody cares about people like me',

'reported being turned away from health facilities', 'did not think it was worth trying to seek care (health care not good enough, health workers' attitudes are bad and thought would get better by oneself)', 'main reason for not using public health facilities', 'what was the main reason that person was unable to get the healthcare they needed?', respondents who indicated 'they have been to public health facilities before and they could not be helped' and 'that the staff are too unfriendly or unhelpful' to the question asked: 'In the past 12 months, was there anybody in this household who needed healthcare but was unable to get it?'

Independent variables

We selected independent variables based on the objective of this study and the review of previous studies [47-49], with consideration of the information available in the 2017–2018 GCRO QoL survey. According to the convention used in earlier studies, the variables were broadly divided into three groups: demographics, economic, and health-related factors [6,33]. Demographic variables assessed in this study included age ('18–19', '20–24' and '25-29'), sex (male* and female), population group (Black African* and Non-Black African), household main spoken language (IsiZulu*, Sesotho, Sepedi and other languages), education (secondary or lower*, matric, and higher) and migration status (in-migrant* and immigrant) [47-49]. Economic variables assessed were employed (no* and yes) and any income (no* and yes). Any income, a proxy for wealth index (socioeconomic status) was derived in the survey through the principal factor of inquiry of available earning accruing over a given period of time (in South African Rands). Health-related factors, including health facility type (private*, public, and both private and public), medical aid cover (yes* and no), health in the last 12 months (excellent*, good, and poor), HIV test in last 12 months (no*, yes, and does not remember), household member HIV status (negative* and positive), disability (no disability* and disabled), mental health condition in the last year (no* and yes) and health satisfaction (satisfied*, dissatisfied, and neither satisfied or dissatisfied) were equally assessed in this study. These variables and their categorization compare well with those of previous studies in South Africa and internationally [50,51]. (Note: * is the reference category used in analyses).

Statistical analysis

Prior to data analysis, the dataset was weighted for under-sampling and over-sampling errors based on past studies [52]. In addition, all data analyses were based on migrant status (in-migrants and immigrants) [33] Univariate analysis was used to describe the characteristics of the study population against each aforementioned explanatory variable using frequency tabulation, while bivariate analysis, which made use of Chi-Square test, was performed and ρ-values were reported to assess the unadjusted associations between the outcome variable and the various explanatory variables by comparing the differences in the proportion of migrants medically excluded between variable categories. To evaluate the adjusted relationship between outcome and the explanatory variables, multivariable binary logistic regression analyses were carried out, accounting for the effects of all other explanatory variables included in the models. To ensure that no important explanatory factors were missed, variables with $\rho \leq 0.20$ in the Chi-Square test were selected for inclusion in the initial multivariable regression model in line with practice in previous studies [53]. This cut-off point was chosen following a critical appraisal of evidence in the literature [31,33]. A logistic binary regression analysis was then performed to obtain the final close models, which only retained explanatory variables significantly associated with the outcome variable at 5% level (ρ-value < 0.05). Unadjusted and adjusted odds ratios in the close by models together with its 95% CI and ρ-values was reported. To reduce possible statistical errors, analyses were cross-checked, and all variables that satisfied inclusion criterion were included in the models. Multicollinearity was checked using 'vif' command and the mean vif was 1.57, and data management was performed using Stata. Furthermore, the fixed effects section of the models was made

up of demographic-level, economic-level and health-related level factors. All statistical analyses was conducted using Stata version 14.0 (StataCorp, USA) with the 'svy' command to adjust for sampling weights, clustering effects and stratification. All the regression analyses results were depicted as odds ratios (OR) at 95% confidence intervals (95% CI).

Results

Socio-demographic Characteristics

Table 1 below shows the demographic, economic and health-related characteristics of non-migrants, in-migrants, and immigrants aged 18-29 years in the Gauteng province of South Africa.

Table1: Socio-demographic, economic and health-related characteristics of non-migrants and migrants in Gauteng, 2017–2018 (N=4 872)

Factors	Non-migrants (n = 2,710)		In-migrants (n = 1725)		Immigrants (n = 437)		Total (N = 4,872)	
	F	%	F	%	F	%	F	%
Demographics characteristics								
**Age**								
18–19	323	11.9	107	6.2	30	7.0	460	9.45
20–24	1050	38.7	650	37.7	133	30.4	1832	37.6
25–29	1338	49.4	968	56.1	273	62.7	2580	53.0
**Sex**								
Male	1294	47.8	853	49.4	239	54.8	2386	49.0
Female	1416	52.2	873	50.6	197	45.2	2486	51.0
**Population group**								
Black African	2410	88.9	1675	97.1	412	94.5	4497	92.3
Non-Black African	300	11.1	50	2.9	24	5.5	375	7.7
**Household main language**								
IsiZulu	835	30.8	439	25.4	77	17.6	1351	27.7
Sesotho	489	18.0	105	6.1	45	10.2	638	13.1
Sepedi	274	10.1	438	25.4	2	0.4	714	14.7
Other languages	1112	41.0	743	43.1	313	71.8	2169	44.5
**Education**								
Secondary and Lower	726	26.8	425	24.7	266	60.9	1417	29.1
Matric	1387	51.2	906	52.5	121	27.8	2414	49.6
Higher	597	22.0	394	22.9	49	11.3	1040	21.4
Economic characteristics								
**Employed in the last week**								
No	2020	74.5	1259	73.0	248	56.9	3528	72.4
Yes	690	25.5	466	27.0	188	43.1	1344	27.6
**Any income**								
No	253	9.3	122	7.1	31	7.1	406	8.3
Yes	2457	90.7	1603	92.9	406	92.9	4466	91.7
Health-related characteristics								
**Medical aid cover**								
Yes	393	14.5	220	12.7	36	8.3	649	13.3
No	2317	85.5	1505	87.3	400	91.7	4223	86.7
**Health in the past 4 weeks**								
Excellent	1296	47.8	753	43.7	177	40.5	2227	45.7
Good	1325	48.9	916	53.1	253	58.0	2494	51.2
Poor	89	3.3	55	3.2	06	1.5	151	3.1
**HIV test in past 12 months**								
No	1887	69.6	1229	71.2	268	61.5	3384	69.5
Yes	735	27.1	412	23.9	147	33.7	1294	26.6
Does not remember	88	3.3	84	4.9	21	4.8	194	3.9
**Household member HIV status**								
Negative	2515	92.8	1635	94.8	424	97.1	4573	93.9
Positive	195	7.2	91	5.2	13	2.9	299	6.1
**Disability**								
No disability	2644	97.5	1699	98.5	431	98.7	4774	98.0
Disabled	67	2.5	26	1.5	06	1.3	98	2.0
**HH Mental health condition**								
No	2501	92.3	1615	93.6	425	97.3	4541	93.2
Yes	210	7.7	110	6.4	12	2.7	331	6.8
**Health services satisfaction**								
Dissatisfied	806	29.7	478	27.7	82	18.7	1365	28.0
Neither	320	11.8	173	10.0	38	8.7	532	10.9

Satisfied	1584	58.5	1074	62.3	317	72.5	2975	61.1

Source: GCRO QoL Survey, 2017–2018

More of the sampled respondents were aged 25-29 years comprising 49.4% of the non-migrants, 56.1% of the in-migrants and 62.7% of the immigrant population. The population of study had more females, in the proportion of 52.2% for the non-migrants and 50.6% of in-migrants. A majority of the respondents of non-migrant (88.9%), in-migrants (97.1%) and immigrants (94.5%) were Black Africans, while a majority of the respondents spoke other languages (Other languages are Afrikaans, Swati, Tsonga, Tswana, Venda, Ndebele, Xhosa and English). While only 51.2% and 52.5% of immigrants and non-immigrants respectively, had completed high school, 60.9% of immigrants had completed secondary school and lower; and a majority of the non-migrant (85.5%), in-migrant (87.3%) and immigrant (91.7%) populations had no medical aid cover (Table 1).

Furthermore, 74.5% of non-migrants, 73.0% of in-migrants and 56.9% of immigrants were not employed in the last week while a majority of the respondents reported having any income (any income' reported by the in-migrants was from the South African Social Security (SASSA) grant paid by the government and the SASSA Grant payment include older persons grant, disability grant and children's grant. The immigrants will generate any income from domestic works to informal jobs with lower wages as they do not have access to SASSA grant payments). Among the groups of respondents, a majority of them reported not having disability, negative HIV status and no mental health conditions among their household members. In addition, more non-migrants (29.7%) than in-migrants (27.7%) and immigrants (18.7%) reported dissatisfaction with health services (Table 1).

Percentage distribution of non-migrants, in-migrants, and immigrants aged 18–29 years

Figure 2 shows the percentage distribution of non-migrants, in-migrants, and immigrants aged 18–29 years in the Gauteng Province in South Africa. A majority of the respondents are non-migrants (55.6%), followed by in-migrants (35.4%) followed by the lowest proportion of immigrants (9.0%) in Gauteng (Fig. 2).

Prevalence of medical exclusion according to migration status

Figure 3 shows the prevalence of medical exclusion according to migration status. From a total population of 2162 migrants, about 5.8% of in-migrants and 4.2% of immigrants reported having been medically excluded from healthcare services.

Percentage distribution of the proportion of migrants and the health facility type utilized

Figure 4 shows the distribution of the proportion of migrants and the health facility type they utilized in Gauteng Province. From the sample size of 2162, more of the sampled in-migrants (84.3%) and immigrants (87.1%) reported the use of a public health facility than other health facility types (private and both health facility types) (Fig. 4).

Percentage distribution of migrants and health service satisfaction

Figure 5 shows the distribution of the proportion of migrants and health service satisfaction in the Gauteng Province of South Africa. In a sample of 1725 in-migrants in the province, 62.3% reported to be satisfied with the health services they receive while 27.7% indicated to be dissatisfied. Among 437 immigrants, 72.5% indicated to be satisfied with health services they receive, and almost 20 percent indicated to be dissatisfied (18.7%) (Fig. 5).

Bivariate analysis of factors associated with medical exclusion among in-migrants and immigrants

From table 2 below, only age, sex, population group, household main language, education, employed in the last week, any income, medical aid cover, health in the past 4 weeks, household mental health condition and health satisfaction showed to be statistically associated with medical exclusion among

in-migrants (ρ<0.05). Among immigrants, only age, sex, population group, household main language, education, employed in the last week, any income, medical aid cover, disability and health satisfaction showed to be statistically significant with medical exclusion among in-migrants (ρ<0.05) (Table 2).

Table 2: Bivariate analysis of factors associated with medical exclusion among in-migrants and immigrants in Gauteng Province of South Africa, 2017–2018 (n = 2162)

Factors	In-migrants					Immigrants				
	Not excluded (n=1 625)		Excluded (n=100)		Chi-square	Not excluded (n=418)		Excluded (n=18)		Chi-square
	F	%	F	%	□2	F	%	F	%	□2
**Demographic characteristics**										
**Age**					0.58**					1.77**
18-19	101	94.1	6	5.9		28	93.6	2	6.4	
20-24	620	95.4	30	4.6		129	96.9	4	3.1	
25-29	904	93.4	64	6.6		261	95.5	12	4.5	
**Sex**					1.89*					0.24*
Male	808	94.8	45	5.2		229	95.9	10	4.1	
Female	817	93.7	55	6.4		189	95.6	9	4.4	
**Population group**					1.28*					2.66*
Black African	1,578	94.2	97	5.8		394	95.5	18	4.5	
Non-Black African	47	93.7	3	6.3		24	100.0	0	0.0	
**Household main language**					0.99					10.23*
IsiZulu	420	95.8	19	4.3		72	93.5	5	6.5	
Sesotho	101	95.8	4	4.2		41	92.6	3	7.4	
Sepedi	410	93.6	28	6.4		2	100.0	0	0.0	
Other languages	695	93.4	49	6.6		303	96.8	10	3.2	
**Education**					7.04***					3.19
Secondary and lower	392	92.3	33	7.7		252	94.7	14	5.3	
Matric	848	93.6	58	6.4		120	98.9	1	1.1	
Higher	385	97.5	10	2.5		46	94.2	3	5.8	
**Economic characteristics**										
**Employed in the last week**					0.98**					0.12**
No	1,185	94.1	75	5.9		240	96.4	9	3.6	
Yes	441	94.5	25	5.5		179	95.0	9	5.1	
**Any income**					1.23*					1.56**
No	119	97.6	3	2.4		29	93.8	2	6.3	
Yes	1,506	93.9	97	6.1		389	95.9	16	4.1	
**Health-related characteristics**										
**Health facility type**					1.91					1.61
Private	122	95.9	5	4.1		38	95.3	2	4.7	
Public	1,367	94.0	88	6.1		364	95.7	17	4.4	
Private and public	137	95.3	7	4.7		17	100.0	0	0.0	
**Medical aid cover**					2.85***					0.25***
Yes	212	96.6	7	3.4		34	94.8	2	5.2	
No	1,413	93.9	93	6.2		384	95.9	17	4.1	
**Health in the past 4 weeks**					15.48**					0.54
Excellent	715	95.0	38	5.0		169	95.5	8	4.5	
Good	863	94.2	53	5.8		243	95.9	10	4.1	
Poor	47	84.1	9	15.9		6	100.0	0	0.0	
**HIV test in last 12 months**					3.98					1.26
No	1,149	93.5	80	6.5		257	95.6	12	4.4	
Yes	395	95.9	17	4.1		140	95.5	7	4.5	
Does not remember	81	96.2	3	3.8		21	100.0	0	0.0	
**Household member HIV status**					27.98*					0.37
Negative	1,551	94.9	84	5.1		406	95.9	1	7.9	
Positive	74	81.8	17	18.2		12	92.1	17	4.1	
**Disability**					1.68					11.19**
No disability	1,599	94.1	100.0	5.9		414	96.1	17	4.0	
Disabled	26	100.0	0	0.0		4	75.9	1	24.1	
**HH Mental health condition**					8.49***					0.65
No	1,527	94.5	88	5.5		407	95.7	18	4.3	
Yes	98	89.3	12	10.7		11	97.7	0	2.3	
**Health services satisfaction**					48.19**					32.66*
Neither	165	95.3	8	4.7		37	97.2	1	2.8	
Dissatisfied	424	88.8	54	11.2		70	85.9	12	14.1	
Satisfied	1,036	96.4	38	3.6		311	98.2	6	1.9	

Source: GCRO, 2017–2018; *ρ < 0.001***, ρ < 0.01**, ρ< 0.05* is considered statistically significant (Chi-Square test).*

For instance, 7.7% of in-migrants with secondary and lower education indicated medical exclusion, while 5.8% of immigrants with higher education reported medical exclusion. Only about 6.0% of the in-migrants not being employed in the last week reported being medically excluded, and 5.1% of the employed immigrants in the last week reported medical exclusion (Table 2). In-migrants with no medical aid cover reported medical exclusion (6.2%), while 5.2% of immigrants with medical aid cover reported medical exclusion. In-migrants with poor health in the past four weeks reported medical exclusion, while 4.5% of immigrants with excellent health in the last four weeks reported medical exclusion. About 10.7% of in-migrants with household mental health condition reported medical exclusion, while 4.3% of immigrants with household mental health condition reported medical exclusion (Table 2). In addition, 11.2% of in-migrants and 14.1% of immigrants who were medically excluded reported dissatisfaction with health services, while 4.7% of in-migrants and 2.8% of immigrants who were medically excluded indicated neither satisfaction nor dissatisfaction with health services, and 3.6% of in-migrants and 1.9% of immigrants who were medically excluded signified satisfaction with health services (Table 2).

The results of the multivariate of the unadjusted and adjusted logistic regression analysis are shown in Table 3. For demographic characteristics, one factor, higher education, is significantly associated with medical exclusion of the migrant population from healthcare services. Similarly, two economic characteristics (employed in the last week and yes to any income), as well as six out of the seven health-related characteristics (no medical cover, poor health in the past four weeks, yes to HIV test in the past 12 months, household member positive HIV status, yes to household mental health condition and dissatisfied with health services) were found to be significantly associated with medical exclusion among migrant populations (Table 3). Specifically, the odds of medical exclusion was more than 10.0% higher among age group cohorts of 25–29 years compared to the age group cohort of 18–19 years (Unadjusted AOR (UAOR) 1.02, 95% CI 0.461–2.278) and 7.0% lower odds of being medically excluded from healthcare services among age group cohorts of 20–24 years compared to their counterparts aged 18–19 years (UAOR 0.71, 95% CI 0.305–1.633). In the adjusted model, age group 20–24 years and 25–29 years have 7% and 9.4% decreased odds of medical exclusion from healthcare services. Female respondents had increased odds of being medically excluded from healthcare services compared to their male counterparts in both UAOR and AOR (Table 3).

The odds of medical exclusion was decreased among non-Black African compared to their Black African counterparts in both the unadjusted and adjusted model. Further, the odds of medical exclusion was increased among migrants who speak Sesotho, Sepedi and other languages compared to their counterparts who speak IsiZulu. Also, the odds of medical exclusion were 8.4% and 7.7% lower among the migrant population with matric education in the UAOR and AOR, respectively; while the odds of medical exclusion were 4% and 3.6% significantly lower among the migrant population with higher education in the UAOR and AOR, respectively (Table 3). For economic factors, the inclusion of respondents who said yes, they have been employed in the last week in the UAOR and AOR showed significantly decreased odds of medical exclusion for the migrant populations. While in the unadjusted and adjusted models, those with any income were significantly associated with lower probabilities of medical exclusion across in-migrant and immigrant populations. Other factors that increased odds of medical exclusion among in-migrants and immigrants were no medical cover, poor and good health in the past 4 weeks, household positive HIV status, household mental health condition and neither or dissatisfied with health services (Table 3).

Table 3: Unadjusted and adjusted logistics regression analysis of predictors of medical exclusion among migrants in Gauteng Province of South Africa, 2017–2018 (n = 2162)

Factors	Unadjusted			Adjusted		
	**Odds**	**95% CI**	**ρ-value**	**Odds**	**95% CI**	**ρ-value**
Demographics characteristics						
Age						
18–19 (RC)						
20–24	0.71	0.305 – 1.633	0.42	0.66	0.269 – 1.622	0.37
25–29	1.02	0.461 – 2.278	0.95	0.94	0.398 – 2.221	0.89
Sex						
Male (RC)						
Female	1.21	0.781 – 1.888	0.39	1.07	0.678 – 1.705	0.76
Population group						
Black African (RC)						
Non-Black African	0.76	0.181 – 3.235	0.72	0.89	0.202 – 3.892	0.87
Household main language						
IsiZulu (RC)						
Sesotho	1.13	0.510 – 2.490	0.77	1.06	0.464 – 2.409	0.89
Sepedi	1.43	0.742 –2.737	0.29	1.42	0.709 – 2.835	0.32
Other languages	1.23	0.745 – 2.032	0.42	1.42	0.840 – 2.395	0.19
Education						
Secondary and Lower (RC)						
Matric	0.84	0.524 – 1.334	0.45	0.77	0.456 – 1.287	0.31
Higher	0.40	0.197 – 0.819	0.01**	0.36	0.163 – 0.815	0.01**
**Economic characteristics**						
Employed in the last week						
No (RC)						
Yes	0.96	0.577 – 1.608	0.00***	0.94	0.556 – 1.603	0.03*
Any income						
No (RC)						
Yes	0.84	0.837 – 4.022	0.01**	0.36	0.971 – 5.730	0.05*
**Health-related characteristics**						
Medical aid cover						
Yes (RC)						
No	1.60	0.702 – 3.652	0.00***	1.23	0.450 – 3.362	0.05*
Health in the past 4 weeks						
Excellent (RC)						
Good	1.11	0.714 – 1.723	0.65	1.09	0.691 – 1.724	0.71
Poor	3.21	1.357 – 7.588	0.01**	1.89	0.745 – 4.805	0.01**
HIV test in past 12 months						
No (RC)						
Yes	0.67	0.375 – 1.193	0.05*	0.74	0.397 – 1.381	0.02*
Does not remember	0.48	0.116 – 2.021	0.32	0.49	0.122 – 1.960	0.31
Household member HIV status						
Negative (RC)						
Positive	3.96	2.181 – 7.203	0.00***	3.67	1.971 – 6.819	0.00***
Disability						
No disability (RC)						
Disabled	0.79	0.161 – 3.868	0.77	0.42	0.087 – 2.045	0.28
HH Mental health condition						
No (RC)						
Yes	1.99	1.024 – 3.869	0.04*	1.55	0.789 – 3.031	0.02*
Health services satisfaction						
Satisfied (RC)						
Neither	1.39	0.563 – 3.425	0.48	1.59	0.606 – 4.174	0.35
Dissatisfied	4.01	2.534 – 6.345	0.00***	4.29	2.528 – 7.270	0.00***

Source*: GCRO Survey, 2017-2018;* Significant p-values: $*\rho \leq 0.05; ** \rho \leq 0.01; *** \rho \leq 0.001$: 95% Confidence intervals (CI); AOR, adjusted odds ratio; UOR, unadjusted odds ratio; RC, Reference Category; Adjustment variables of the multivariable models are age, marital status, educational level, residence, work status, wealth quintile, and provinces.

Discussion

This study assessed the health services satisfaction and medical exclusion among migrant youths, using the GCRO survey (2017–2018) in Gauteng Province, South Africa. In order to facilitate universal health coverage (UHC) for migrants, or to reduce exclusion, inequities, and discrimination against migrants, it is crucial to prioritize the healthcare needs of migrant youths. The World Health Organization (WHO) definition of UHC was employed and this embodies the strategic concepts of non-discrimination, quality, and financial risk protection [4,54]. Although there are controversies surrounding National Health Insurance (NHI) in South Africa [55,56], the 2019 NHI Bill is a significant indicator for the goals of the South African government to make available UHC for in-migrants and immigrants. In most cases, the incomprehensible thing about South Africa legislation and policies show that they equally expedite and exclude healthcare services for migrants, especially immigrant populations. Despite the existence of rights-based, non-discriminatory legislation and other forms of legal authority or sanctions of the National Health enactment, the legal documents codifying the discussions on the legal status of migrants as the most important determinant of their healthcare access are the immigration statutes and the 2019 NIH Bill [27,57].

In South Africa, the act of exclusion from access to healthcare and satisfaction with health services among migrants is aggravated by disparities in communicating the final outcome of the subject matter, in the clarification and interpretation, content, and/or implementation and enforcement of the recommended outcome policies at both at the provincial and national levels. For instance, Lenano [58] has emphasized the unpredictable changes and unstable nature of policies and strategic interventions that are insensitive to migrants' health needs across Europe and the USA [59]. According to White et al. [45], the sense of migrants' moral worth or meritoriousness to obtain high-quality medical care is a factor of social welfare and health policies of entitlement or exclusion. Also, several studies have illustrated the influence of legal status on migrants' access to all-inclusive health services [42]. The WHO and the International Organization for Migration (IOM) have undeniably stated that meeting the social and health needs of migrants, especially immigrants, asylum seekers, and refugees by ensuring that they have access to high-quality and affordably priced health-care services, is necessary to achieving the SDG target on UHC [60,61].

Our study showed a prevalence of 55.6% of non-migrants, 35.4% of in-migrants and 9.0% of immigrants aged 18–29 years in the Gauteng Province in South Africa. This result is higher than the studies conducted at the national level, which showed that only 3% – 4% of people in South Africa are from outside of the country [62]. Also, the prevalence of migrants in this study's findings showed that it is much lower than the prevalence in studies conducted by Statistics South Africa [36] and Mukumbang et al. [4], which reported more than 50% of migrant populations residing in South Africa. In a similar line, the study's findings are lower than those of a survey done in the Southern African Development Community [63], but higher than those of a study done in South Africa, which found a 5% prevalence of migrants with the same characteristics [42]. This study also revealed a prevalence of 6% of in-migrants and 4.2% of immigrant who reported medical exclusion from healthcare services. Thus, this study's findings further showed that about 10% of migrants experienced medical exclusion, with a low prevalence (4.2%) of medical exclusion reported among immigrants. The finding was much lower than other studies conducted in South Africa (20.0% and 48%) [45,51]. The outcomes were significantly lower than those of research studies conducted in Ghana [64,65], as well as those conducted in Australia [15] and in Europe [64] combined. The discrepancy might be owing to variations in the study period, that is, Gauteng City-Region Observatory study use GCRO 2017–2018 datasets, while cited studies across Europe and Africa were conducted in 2013, 2018 and 2022.

The disaggregated datasets were stratified by migrant status, and further analysis was carried out to determine the types of health facilities they use. The results indicated a majority of migrant populations (immigrants – 87.1% and in-migrants – 84.3%) reporting the use of public health facilities rather than other health facilities combined (both private and public). The use of healthcare services varies significantly by migrant status, and in South Africa, documented migrant youths are entitled to free medical care to an extent [62,66]. Studies have shown that immigrants who go to public health facilities will be means-tested in order to check if they qualify for free healthcare and many immigrants have narrated their worst experiences on unfair health treatment they received from healthcare providers [67,68]. For instance, some immigrants in South Africa reported that their health needs are falling, as these were compounded during the lockdown since they were refused healthcare, and more apprehensions were evident that they might be excluded from the vaccine rollout [69]. Our study also showed that more immigrants than in-migrants reported health services satisfaction as well as dissatisfaction of health services. In contrast, a South African study found that immigrants were less satisfied with the health services they received [70-72]. Similarly, immigrants who reported satisfaction with health services have described uncaring attitudes and perceptions of discrimination when accessing healthcare for the first time. In other studies conducted by Winters et al. [71] and Allegri et al. [72], the uncaring attitudes of health workers towards immigrants pose a huge barrier for exclusion from health care services and a danger to other population groups, especially among immigrants with chronic health conditions such as HIV and TB infections [73,74].

Among the selected co-variates, demographic characteristics (such as age of respondents as 25-29 years), sex (female) and household main language (Sesotho, Sepedi and other languages)), and health-related characteristics (such as no medical aid cover , health in the past four weeks (good and poor), household member HIV status (positive), household mental health condition (yes), and health services satisfaction (neither satisfied nor dissatisfied) were the major predictors of medical exclusion among migrants in the unadjusted and adjusted logistics regression analysis. We also found a non-significant relationship between age of respondents and medical exclusion among migrants in South Africa. The unadjusted odds ratio showed that respondents who are aged 25-29 years were more likely to be medically excluded from health care services compared to those who are aged 18-19 years. This could be due to the stigmatization that goes with age group of 25-29 years, that they are the major migrant populations that are over-burdening the health care facilities in South Africa. Studies of a similar nature conducted in sub-Saharan Africa demonstrated that as migrants aged, they were more likely to be denied access to medical care services [75-77].

Our study revealed that, compared to their male counterparts, female migrants were more likely to be denied access to healthcare services. This clearly illustrates the gender gap in accessing healthcare facilities. Women encounter many healthcare hurdles, which prevents them from easily accessing and acquiring the necessary care. A recent report revealing how a Limpopo Provincial Health Minister reprimanded a Zimbabwean female migrant who was seeking medical treatment in South Africa, leading to a protest against medical xenophobia [66,67]. Another well-cited instance indicated how a South African advocacy group for the immigrant population launched court proceedings against an upfront fee of an estimated 935 US Dollars for maternity cases, and 3000 US Dollars for routine surgical cases for female migrants, while non-migrants with such cases have free access to all aforementioned treatment [66,67]. Migrants who speak *Sesotho*, *Sepedi* and other languages (Other languages are *Afrikaans, Swati, Tsonga, Tswana, Venda, Ndebele, Xhosa* and English) as their household main languages were more likely to be excluded from health care services compared to those who speak *IsiZulu* as their household main language. This finding is based on the spoken languages in South

Africa, as at least thirty-five languages are indigenous to South Africa, and eleven of which are official: *Zulu* (*isiZulu*), *Pedi* (*Sepedi*), *Sotho* (*Sesotho*), *Afrikaans*, *siSwati*, *Xitsonga*, *Setswana*, *Tshivenda*, *Ndebele*, *isiXhosa*, and English, which is the primary language used in parliamentary and state discourse, though all official languages have equal legal status [78]. Several historical studies have showed the multilingualism and *polylectalism* in these household languages [78-80]. For instance, the language *Sepedi* or *Pedi* is a *Sotho-Tswana* language spoken as the fifth language in South Africa and is native to Lesotho and Zimbabwe. The language is mostly spoken in Mpumalanga, Gauteng and the Limpopo provinces of South Africa.

The *Sesotho* language is also one of the two official languages of Lesotho, one of the sixteen official languages of Zimbabwe, and one of the eleven official languages of South Africa. Other South Africans can be found speaking it in all the main residential regions of Metropolitan Municipalities like Johannesburg and *Tshwane* [81]. Additionally, *Zulu* is a southern *Bantu* language of the Nguni branch spoken in Southern Africa and is the native tongue of the Zulu people, who are mainly found in the South African region of KwaZulu-Natal [80-81]. *Zulu* is the most widely spoken language in South Africa (46% of the population) and the most widely used first language (23% of the population). Particularly in Zimbabwe, and the main dialect *Xhosa* in the Eastern Cape, where the Northern *Ndebele* language (isiNdebele) is closely connected to *Zulu* (Maho (2009) lists four dialects: Central KwaZulu-Natal, Northern Transvaal *Zulu*, Eastern Coastal *Qwabe*, and Western Coastal Cele (Reference). Standard *Zulu* also called “deep *Zulu*” is taught in schools in South Africa), the migrating *Zulu* inhabitants have had their spoken language diffused among the surrounding regions/provinces [82]. Drawing inferences from above clarifications on the household main languages, migrants who speak these South African official languages that are native to other neighbouring countries might be medically excluded, as the nationals will know that some of these patients are foreigners (migrants) [75,76]. In the same vein, when undocumented migrants cannot provide a residency permit, they are not allowed to have access to basic medical healthcare.

We also found a significant relationship between the economic characteristics and medical exclusion among migrants in both unadjusted and adjusted odds ratio. Our study showed that migrants who were employed in the last week were less likely to be medically excluded from healthcare services in both models, while migrants with any income were found less likely to be medically excluded from healthcare services in both models. This aligns with a study conducted in South Africa that migrants pay for their healthcare services just as South African nationals do [66]. Thus, non-South Africans are either subject to the same means-tested hospital fees, or they are subject to the highest fees if they are undocumented and not from the Southern African Development Community (SADC) [63,45] and this was also shown in a recent study conducted in Europe [64,83]. Consequently, the out-of-pocket payment system for healthcare services is a significant obstacle for unemployed migrants who lack any means of supplemental income. This typically will have an impact on the most vulnerable and destitute migrants who cannot afford to pay for their medical expenses [84,85].

Regarding the health-related characteristics, we found that the odds of migrants who were medically excluded were higher among those with no medical aid cover in both models. Studies based on migrants who did not have medical aid, thereby experiencing medical exclusion, had reported similar findings [84,85]. Similarly, migrants with poor or good health in the past 4 weeks had higher odds of being medically excluded from healthcare services in both unadjusted and adjusted models. A possible explanation could be the two health-related characteristics at migrants awaiting clarification of their status and those without documentation will experience some forms of medical xenophobia [67,85]. The findings show that the odds of migrants who were medically excluded decreases with those who

had HIV test in the past 12 months. Given that South Africa has an estimated TB incidence of 860/100,000 and an estimated HIV + TB co-infection of 520/100,000, this could be one explanation for the lack of attention given to migrants living with HIV/TB or/and co-infections [86]. Despite this, TB is still the biggest cause of death for people with HIV in South Africa, and the rise of drug-resistant TB strains, which are more challenging and expensive to treat, has made the situation even worse. In order to expedite the start of antiretroviral therapy (ART) for PLHIV with TB, the Centers for Disease Control and Prevention (CDC) in South Africa, through its Global AIDS Program (GAP), is collaborating closely with the NDoH, much like with Covid-19, to strengthen HIV/TB screening for all persons living with HIV (PLHIV), including migrants [87].

The study also suggests that a household member with a positive HIV status predicts medical exclusion of migrants from healthcare services. In the unadjusted and adjusted models, migrants who had a household member with a positive HIV status were 4.0 and 3.7 times more likely to experience medical xenophobia, respectively. Studies similar to this one conducted in Mozambique [88] and South Africa [89] reported conclusions that were similar. The possible explanation could be that a there is a gap in literature on barriers to migrants reporting their household member with an HIV-positive status or engaging healthcare systems with such medical problems, as shifting in cultural and clinical settings ranging in structural vulnerabilities that are limiting immigrants' household members from accessing and having proper integration within healthcare services. These barriers include stigmatization of HIV-positive household members, social seclusion, xenophobia and deportation, marginalization and mistreatment, language obstacles, ethnic hostility, and medical heterogeneity [88,89]. Simultaneously, these barriers may lead to medical exclusion, deferment of treatment-seeking and deterring drug adherence, which could escalate proportions of indisposition and death as well as promoting viral mutation and antiretroviral drug resistance [90].

Our study findings also suggest that more migrants with a household member with a mental health condition were reported to have experienced medical exclusion from health services compared to those who do not have a household member with a mental health conditions. Some studies have reported that immigrants with a household member with mental health challenges could face experienced, anticipated, and internalized stigmas, from stereotyping and prejudice, to discriminatory attitudes from healthcare providers [84,85], and so will not be able to communicate their household health problems to the public. Hence, we found that the predictors for migrants who were neither satisfied nor dissatisfied with health services were reported to be medically excluded from health care services. Similar studies conducted in Lesotho [87] and in Zimbabwe [90] have reported results similar to this study's findings. The possible explanation could be that migrants who were excluded from the health facilities initially may report high rates of being neither satisfied nor dissatisfied with the health services and health providers [88].

Implications of findings on medical exclusion of migrants in South Africa

In South Africa, it is estimated that about four million migrants are residing within and outside the borders of South Africa, and this phenomenon is accompanied by a history of exploitative labour arrangements between South Africa and its neighbouring nations. This past is frequently portrayed with a 'historical amnesia' of migrants' contributions to the economy and society of South Africa. Most migrants, and particularly African immigrants, are treated with distrust and hostility known as xenophobia. This is in contrast to their in-migrant counterparts, who are South Africans who have moved from one province to another in search of better educational and economic opportunities [91,92]. This prejudice toward immigrants is mirrored in South Africa's public health system, and this approach has not addressed the realities involved with migration and health, nor does it meet the needs of

immigrants. The South African Immigration Act is silent on the health rights and needs of immigrants, placing them in a vulnerable situation and often leading to their exclusion from the public health system; subsequently, undocumented immigrants are not legally recognized, and their situation is exacerbated [62,92].

In contrast, recent media reports have often focused on 'how an influx of health migrants' has placed a strain on the country's ability to deliver health care to its nationals. Several provincial health departments have expressed concern about a lack of resources due to immigrants' demands [66,91]. The media reports and official verdicts have created conditions that deny refugees, asylum seekers, and undocumented immigrants access to public clinics and hospitals. Denying migrants access to health care constitutes a violation of the internationally recognized right to access healthcare services. The law has also been enshrined in the national constitution of South Africa [66,68]. All immigrants are entitled to these constitutional rights, including the right to life, dignity, freedom, and security, as well as the right to information and just administrative acts. Section 27 of the Constitution guarantees everyone the right to basic health care, affirming that "everyone has the right to have access to health care services, including reproductive health care" and that "no one may be refused emergency medical treatment" [66,69].

The violation of migrants' rights to access health care has grave consequences, having a gendered, racial and class influence, with poor and economically disadvantaged immigrants bearing the burden of this discrimination [62,67]. The provision of health care services to migrants is justified on grounds related to public health in addition to the state's legal obligation to offer access to healthcare. Undocumented immigrants, refugees, and those seeking asylum may have been exposed to communicable diseases during their long journey to South Africa from their home countries. We live in a common social space, so treating these illnesses is in the public's best interest. Given the migrant population's absorption into the larger community, the health of the local population is correlated with the migrant population's health. In order to tackle migration and health beyond infectious diseases and border checks, the South African government and pertinent health stakeholders should establish a comprehensive multi-sectoral approach. Both the National Health Act and the Immigration Act should explicitly provide for migrant health care. The Immigration Act must be revised to properly recognize the rights to healthcare of both documented and undocumented immigrants [66,69]. The law should be supplemented by an extensive national strategy that specifies how undocumented migrants should be handled and it should be administered uniformly throughout all provinces.

Also, to enforce migrant health rights, we must speak up and educate health professionals. Such training, developed in collaboration with the South African Department of Health and the Health Professions Council of South Africa, ought to raise and sensitize the awareness of rights and requirements for health care for migrants among medical practitioners. Health administrators should be a part of it as well, as they serve as a point of entry for immigrants trying to receive medical services [67,92]. These measures are necessary, as a public healthcare system that excludes migrants creates conditions for poor public health for all. It increases the vulnerability of migrants, generates and magnifies discrimination and inequalities in health, and violates migrants' constitutional rights to access health care. Furthermore, this is not just a health and human rights issue but it is also a matter of social justice. In South Africa's society and economy, migrant labour has played a crucial role, and their lower wages have increased consumer and business profitability and saved consumers money. Hence, delivering equitable access to care for migrants can reduce the health and social costs of disease, improve social cohesion, protect public health and human rights, and contribute to healthier migrants in healthier local communities.

Strengths and limitations

This study presents a snapshot of medical exclusion and satisfaction rates of health services among migrant youths in Gauteng province of South Africa. To the best of our knowledge, this is the first study to investigate health services' satisfaction and medical exclusion among youth migrants using nationally representative data in South Africa. National representativeness, high response, application of complex sample statistics in all analyses (to adjust for sample weights and cluster design of the survey) and low missing data are some of the strengths of this study. Others include large sample size and the use of a migrant status data disaggregation method. A methodological strength is the combination of univariate, bivariate and multivariate analyses to explore whether medical exclusion from healthcare services can facilitate satisfaction of migrants by status exacerbates exclusion for migrants in South Africa. Regarding the study's limitations, due to the cross-sectional nature of its design, it is possible that the findings will not prove a true causal relationship between the independent variables and the outcome variable. The respondents' self-reports from the two years prior to the 2017–2018 GCRO survey were used to compile the data, which raises the possibility of recall bias and misclassification bias. The low prevalence of immigrant populations, especially for youth cohorts, remains less visible, yet a reflection of a typical pattern of temporary migration (migrant worker) remains insufficiently understood. More research is needed to confirm and explore this topic, hence future studies may consider addressing this limitation.

Conclusion

This paper, based on the data from the fifth round of the GCRO, contributes new evidence to improve our understanding of the health services satisfaction and association with medical exclusion through the analysis of socio-demographic, economic and health-related determinants amongst in-migrants and immigrants in South Africa. In-migrants and immigrants in the study population were found to have a higher prevalence (10.0%) of medical exclusion of migrants (in-migrants – 5.8% and immigrants – 4.2%), while the majority of in-migrants and immigrants have used public health facility types, and about 65.2% of migrants have low satisfaction (in-migrants – 37.8% and immigrant – 27.4%) of health services. These findings, which will be enhanced in future longitudinal follow-up rounds, offer important insights into how migrant youths interface with health service satisfaction in a transitioning context such as South Africa. As such, the study assists in providing evidence to support the development of health and social policies to provide effective healthcare for in-migrants and immigrants as part of developing locally-adapted approaches to universal health coverage. Achieving the intent of the legal, political and health-systems development processes underway concerning National Health Insurance in South Africa can be greatly enhanced by recognizing the as-yet-unmet needs of South Africa's sizeable migrant community. However, legislative changes need to be accompanied by investment in the South African health system, strategies to improve its performance, value-based leadership and management of the health system, and a broad coalition that ensures the prevention and mitigation of medical exclusion of migrants from healthcare services.

Recommendation

Based on the data and findings, the authors provide the following recommendations: first, our findings call for the need to implement health programmes among migrants in South Africa to increase awareness of the negative implications of medically excluding migrants from health services, in order to avoid health risks of morbidity complications and mortality. Second, health legislation and policies should be formed to focus and shape health insurance coverage and, ultimately, access to and utilization of healthcare services among migrants in South Africa. Third, there should be an urgent need for

revision of an enabling constitution, national health care act, and an exclusionary immigration act, as well as an NHI bill in order to ensure an inclusive healthcare system for all people, regardless of their nationality, language spoken, and social status. Fourth, South Africa should work to develop a national migration and health-coordinating network and policy, by drawing on existing policy processes at the local and national level, and in consultation with multiple stakeholders. Findings from this study may be useful in informing policy-makers and public health experts in the area so as to improve the health outcomes of migrants in the area by improving the utilization of health facilities, especially among female migrants and those vulnerable migrants with chronic health conditions such as HIV and mental health issues.

Supplementary information

The online version contains supplementary material and the Do-file for the analysis is uploaded at the time of submission (DOCX).

Acknowledgments

The authors are grateful to GCRO surveys for proving them with the access to the data set. We are also thankful to Mrs. Helen Thomas for her support in language editing

Author Contributions

Conceptualization: Salmon Likoko, Monica Ewomazino Akokuwebe
Data curation: Salmon Likoko, Monica Ewomazino Akokuwebe
Formal analysis: Salmon Likoko, Monica Ewomazino Akokuwebe
Investigation: Monica Ewomazino Akokuwebe, Salmon Likoko
Methodology: Monica Ewomazino Akokuwebe, Salmon Likoko
Research administration: Monica Ewomazino Akokuwebe
Software: Salmon Likoko
Supervision: Monica Ewomazino Akokuwebe, Godswill Nwabuisi Osuafor, Erhabor Sunday Idemudia
Writing – original draft: Monica Ewomazino Akokuwebe
Writing – review editing: Monica Ewomazino Akokuwebe, Salmon Likoko
Payment of Article Publication Charges: Godswill Nwabuisi Osuafor

References

1. International Organization for Migration (IOM) [Internet] *World Migration Report 2020*. Mcauliffe, M. And B. Khadria (Eds.). Geneva. Iom.int. 2019 [cited 2022 August 14]. Available from: https://www.World Migrat ion Report 2020 | IOM Publications Platform.
2. Reliefweb [Internet], 2021. South Africa reckons with its status as a top immigration destination, apartheid history and economic challenges. Reliefweb.int [cited 2022 August 24]. Available from: https://www.reliefweb.int/report/south-africa/south-africa-reckons-its-status-top-immigration-destination-apartheid history.
3. United Nations High Commissioner for Refugees (UNHCR), 2021. South Africa−UNHCR, the UN Refugee Agency. unhcr.org [cited 2022 August 24]. Available from: https://www.unhcr.org/South-africa.html
4. Mukumbang FC, Ambe AN, Adebiyi BO. Unspoken inequality: how Covid-19 has exacerbated existing vulnerabilities of asylum-seekers, refugees, and undocumented migrants in South Africa. Int J Equity Health 2020 Aug; 19: 141.
5. International Labour Organization (ILO) [Internet]. ILO Global estimates on International migrant workers: results and methodology. International Labour Office Geneva. Labour Migration Branch Conditions of Work & Equality Department and Department of Statistics. Pp. 1-74. Ilo.org 2021 [cited 2022 August 14]. Available from: https://www.ilo.org/wcmsp5/groups/public/@dgreports/@dcomm/@publ/documents/publication/wcms_808935.pdf

6. Moultrie T, Dorrington R, Budlender D. Migration in South Africa An analysis of the 2011 South African census data. Johannesburg: African Centre for Migration & Society, University of the Witwatersrand. 2016. Available from: https://www. wits.ac.za/acms/
7. Statistics South Africa (Statistics SA) [Internet]. Census 2022 - Migration. Department: Statistics South Africa, Republic of South Africa, 2021. statssa.gov [cited 2022 August 14]. Available from https://www. statssa.gov.za/?cat=20
8. Vearey J. Healthy migration: a public health and development imperative for South (ern) Africa. South Afr Med J 2014 July; 104 (10): 663-664.
9. Vearey J, Modisenyane M, Hunter-Adams J. Towards migration–aware responses to health in South Africa: an opportunity to address inequity in health? South Afr Health Rev 2017 Dec; 2017 (1): 89-98.
10. Mo Ibrahim Foundation [Internet]. Africa's youth jobs or migration? Demography, economic prospects and mobility. 2019 Ibrahim Forum Report [cited 2022 June 4]. Available from: https://mo.Ibrahim. foundation/ sites/default/files/2020-05/2019-forum-report_0.pdf
11. National Labour Migration Policy (NLMP) [Internet]. Draft National Labour Migration Policy for South Africa, 2022. Department of Employment and Labour, Republic of South Africa. Pp. 1-115. Available from: National Labour Migration Policy (NLMP); Pp. 1-115. Draft National Labour Migration Policy for South Africa. Department of Employment and Labour, Republic of South Africa [cited 2022 August 10]. Available from: https://www.labour.gov.za/DocumentCenter/Publicationns /Public%20Employment%20Services/National%0Labour%20Migration%20Policy%202021%202.pdf
12. Changwe Nshimbi C, Fioramonti L. The Will to Integrate: South Africa's Responses to Regional Migration from the SADC Region. African Development Review, 2014 March; 26(S1): 52–63.
13. Cirillo M, Cattaneo A, Miller A, Sadiddin A. Establishing the link between internal and international migration: Evidence from sub-Saharan Africa. World Development 2022 Sept; 157 (10): 105943.
14. Statistics South Africa. Census 2011: Migration dynamics in South Africa (No. 03-01-79). Statistics South Africa, 2015. Pretoria. Available from: https://www.statssa.gov.za/publications/Report-03-01-79 /Report-03-01-792011.pdf
15. Polonsky MJ, Ferdous AS, Renzaho Andre MN, Waters N, McQuilten Z. Factors leading to healthcare exclusion among African refugees in Australia. Journal of Public Policy & Marketing 2018 Nov; 37 (2): 306-342.
16. Lu Y, Treiman DJ. Migration, remittances, and educational stratification among blacks in Apartheid and post-apartheid South Africa. Soc Forces, 2011 Aug; 89 (4): 1119-1143.
17. Nzabamwita J. African migrants' characteristics and remittance behaviour: empirical evidence from Cape Town in South Africa. African Human Mobility Review 2018 Aug; 4 (2): 1226-1254.
18. World Health Organization (WHO) [Internet]. World Health Organization, 2017. Human rights and health [cited 2022 August 11]. Available from: https://www.who.int/news-room/fact-sheets/detail/human-rights-and-health.
19. The South African Human Rights Commission (SAHRC) [Internet]. SAHRC, nd. Access to health care [cited 2022 August 10]. Available from: https://www.who.int/news-room/fact-sheets/detail/human-rights-and-health.
20. Perreira KM, Crosnoe R, Fortuny K, Pedroza JM, Ulvestad K, Weiland C, Yoshikawa H, Chaudry A. Barriers to immigrants' access to health and human services programmes. Assistant Secretary for Planning and Evaluation (ASPE) Research Brief, 2012 May; Pp. 1-19. Available from: https://aspe.hhs. gov/reports/barriers-immigrants-access-health-human-services-programs-0.
21. Sithole S, Dunbabo MF. Exploring youth migration and the food security nexus: Zimbabwean youths in Cape Town, South Africa. African Human Mobility Review (AHMR) 2016 May-Aug; 2 (2): 512-537.
22. Majee W, Dinbabo MF, Ile I, Belebema M. African immigrant and refugee families' perceptions on informational support and health status: a comparison of African immigrants living in South Africa and the United States. African Human Mobility Review, 2019 Dec; 5 (3): 1696-1716.
23. Abubakar I, Aldridge RW, Devakumar D, Orcutt M, Burns R, Barreto MI, Dhavian P, Fouad FM, Groce N, Guo Y, Hargreaves S, Knipper M, Miranda JJ, Madise N, Kumar B, Mosca D, McGovern T, Rubenstein L, Sammonds P, Sawyer SM, Sheikh K, Tollman S, Spiegel P, Zimmerman C. The UCL-Lancet Commission on Migration and Health: The health of a world on the move. Lancet 2018 Dec; 392 (10164): 2606-2654.
24. Larchanché S. Intangible obstacles: health implications of stigmatization, structural violence, and fear among undocumented immigrants in France. Soc Sci Med 2012 Mar; 74 (6): 858-863.

25. Walls HL, Vearey J, Modisenyane M, Candice M, Chetty-Makkan CM, Charalambous S, Smith RD, Hanefeld J. Understanding healthcare and population mobility in Southern Africa: the case of South Africa. South Afr Med J 2016 Nov; 106 (1): 14-15.
26. Zihindula G, Meyer-Weitz A, Akintola O. Lived experiences of Democratic Republic of Congo refugees facing medical xenophobia in Durban, South Africa. J Asian Afr Stud 2015 Aug; 52 (4): 458-470.
27. Vanyoro KP. 'When they come, we don't send them back': counter-narratives of 'medical xenophobia' in South Africa's public health care system. Palgrave Commun. 2019 Sept; 5 (101): 1-12.
28. Crush J, Tawodzera G. Medical xenophobia and Zimbabwean migrant access to public health services in South Africa. J Ethn Migr Stud 2014 Oct; 40 (4): 655-670.
29. Castelli F, Sulis G. Migration and infectious diseases. Clinical microbiology and infection: the official publication of the European Society of Clinical Microbiology and Infectious Diseases 2017 May; 23 (5): 283-289.
30. Vignier N, Bouchaud O. Travel, migration and emerging infectious diseases. Electronic Journal of the International Federation of Clinical Chemistry and Laboratory Medicine (EJIFCC) 2018 Nov; 29 (3): 175-179.
31. Agyemang C, van den Born B-J. Non-communicable diseases in migrants: an expert review. Journal of Travel Medicine, 2019 Feb; 26 (2): tay 107.
32. World Health Organization (WHO) [Internet]. Prevention and control of non-communicable diseases in refugees and migrants. Copenhagen: WHO Regional Office for Europe, 2019 (Technical guidance on refugee and migrant health) [cited 2022 August 10]. Available from: https://apps.who.int/iris/bitstream/handle/10665/311461/9789289053990-eng.pdf?sequence=1&isAllowed=y
33. Ajaero CK, De Wet-Billings N, Atama C, Agwu P, Eze EJ. The prevalence and contextual correlates of non-communicable diseases among inter provincial migrants and non-migrants in South Africa. BMC Public Health 2021 May; 21, 999.
34. Statistics South Africa (Stat SA) [Internet]. Statistics South Africa releases the mid-year population estimates: yearly archives 2022 [cited 2022 October 6]. Available from: https://www.statssa.gov.za/?p=15589
35. Galal S. [Internet]. Total number of households of South Africa in 2021, by province (in 1,000s). Statista 2022 [cited 2022 October 6]. Available fromhttps://www.statista.com/statistics/ 1112634/number-of-households-of-south-africa-by-province/
36. Statistics South Africa (Stat SA) [Internet]. Mid-year Population Estimates: 2021. Pretoria: Statistics South Africa [cited 2022 October 6]. Available from: http://www.statssa.gov.za/publications/P0302/Mid%20year%20estimates%202021_presentation.pdf
37. Greyling T. An analysis of the quality of life of migrants in Gauteng, a province of South Africa. Journal of Economic and Financial Sciences 2015 July; 8 (28): 495-517.
38. Nhamo L, Rwizi L, Mpandeli S, Botai O, Magidi J, Tazvinga H, Sobratee N, Liphadzi SM, Naidoo D, Modi A, Slotow R, Mabhaudhi T. Urban nexus and transformative pathways towards a resilient Gauteng city-region, South Africa. Cities 2021 Sept; 116: 103266.
39. Industrial Development Corporation (IDC) [Internet]. Northern Cape | Western Cape| Free State | North-West | Limpopo | Mpumalanga | KwaZulu-Natal | Gauteng | Eastern Cape: GAUTENG. Industrial Development Corporation (IDC), 2022 [cited 2022 October 6]. Available from: https://www.idc.co.za/our-regions/gauteng/
40. Writer S [Internet]. Data shows migration patterns in South Africa – with people flocking to Gauteng and the Western Cape. Business Tech, 2021 [cited 2022 August 2]. Available from: https://www.businesstech.co.za/news/lifestyle/507000/data-shows-migration-patterns-in-south-africa-with-people-flocking-to-gauteng-and-the-western-cape/
41. Amnesty International [Internet]. South Africa: living in Limbo: Rights of Asylum Seekers Denied. Johannesburg: Amnesty International, 2019 [cited 2022 October 6]. Available from: https://www.amnesty.org/ en/documents/afr53/0983/2019/en/
42. Moyo K [Internet]. South Africa reckons with its status as a top immigration destination, apartheid history, and economic challenges. Migration Information Source, Migration Policy Institute, 2021 [cited 2022 August 24]. Available from: https://www.migrationpolicy.org/article/south-africa-immigration-desti nation-history
43. Statistics South Africa [Internet]. Documented immigrants in South Africa 2011. Discussion document D0351.4, Statistics South Africa, 2012 [cited 2022 August 2]. Available from: https://www.statssa.gov.za/publications/D03514/D035142011.pdf

44. The Gauteng City Region Observatory (GCRO)'s Quality of Life (QoL) Survey [Internet]. Quality of Life Survey V (2017/2018). Findings from the GCRO's Quality of Life Survey 5 (2017/2018). GCRO Data Brief No. 11. Gauteng City-Region Observatory, November 2018 [cited 2022 August 27]. Available from: https://gcro.ac .za/ research/ project/detail/quality-of-life-survey-v-201718/.
45. White JA, Blaauw D, Rispel LC. Social exclusion and the perspectives of health care providers on migrants in Gauteng public health facilities, South Africa. PloS ONE 2020 Dec; 15 (12): e0244080.
46. White JA, Rispel LC. Policy exclusion or confusion? Perspectives on universal health coverage for migrants and refugees in South Africa. Health Policy and Planning 2021 Oct; 36 (8): 1292-1306.
47. Ajaero CK, Odimegwu CO, Chisumpa V, Obisie-Nmehielle N. The influence of internal migration on mental health status in South Africa. International Journal of Mental Health Promotion 2017 June; 19 (4): 189-201.
48. Akokuwebe ME, Idemudia ES. A Community Study of the Risk Factors and Perceived Susceptibility to Kidney Disease Risk in Lagos State, South West Nigeria. African Journal of Biomedical Research 2022 May; 25 (2): 153-161.
49. Akokuwebe ME, Idemudia ES. Knowledge and Risk Perceptions of Chronic Kidney Disease Risk Factors among Women of Childbearing Age in Lagos State, Nigeria: From a Health Demography Approach. International Journal of Nephrology, 2022 May; 5511555: 1-16.
50. Müllerschon J, Koschollek C, Santos-Hövener C, Kuehne A, Müller-Nordhorn J, Bremer V. Impact of health insurance status among migrants from sub-Saharan Africa on access to health care and HIV testing in Germany: a participatory cross-sectional survey. BMC Int Health Hum Rights 2019 March; 19 (10): 1-13.
51. Ginsburg C, Collinson MA, Gómez-Oliveé FX, Gross M, Harawa S, Lurie MN, Mukondwa K, Pheiffer CF, Tollman S, Wang R, White MJ. Internal migration and health in South Africa: determinants of health care utilization in a young adult cohort. BMC Public Health 2021 March; 21 (1): 554.
52. Fujiwara K, Huang Y, Hori K, Nishioji K, Kobayashi M, Kamaguchi M, Kano M. Over- and under-sampling approach for extremely imbalanced and small minority data problem in health record analysis. Front Public Health May, 2020; 8: 178.
53. Xie Y, Guo Q, Meng Y. The health service use of aged rural-to-urban migrant workers in different types of cities in China. BMC Health Serv Res 2021 June; 21: 606.
54. World Health Organization (WHO) [Internet]. Universal Health Coverage: Moving Towards Better Health Action Framework for the Western Pacific Region. WHO Regional Office for the Western Pacific. World Health Organization, 2016 [cited 2022 August 17]. Available from: https://apps.who.int/iris/handle/10665/246420
55. Weir-Smith G, Mokhele T, Dlamini S. National health insurance in the face of COVID-19: urban tendencies in South Africa. South African Geographical Journal, 2022 March; 104 (3): 276-290.
56. Pauw T. Catching up with the constitution: An analysis of National Health Insurance in South Africa post-apartheid. Development Southern Africa, 2021, ahead-of-print, 1-14.
57. Moyo P, Botha J, Govindjee A. The Constitutionality of the National Health Insurance Bill: The Treatment of Asylum Seekers. Potchefstroom Electronic Law Journal 2022 April; 25: 1 – 30.
58. Lenano A, Hamed S, Bradby H, Gil-Salmerón A, Durá-Ferrandis E, Garcés-Ferrer J, Azzedine F, Riza E, Karnaki P, Zota D, Linos A. Migrants' and refugees' health status and healthcare in Europe: a scoping literature review. *BMC Public Health* 2020 June; 20 (1): 1039.
59. Matlin SA, Depoux A, Schütte S, Flahault A, Saso L. Migrants' and refugees' health: towards an agenda of solutions. *Public Health Rev* 2018 Sept; 39: 27.
60. Loganathan T, Chan ZX, Pocock NS. Healthcare financing and social protection policies for migrant workers in Malaysia. PLoS One, 2020 Dec; 9, 15 (12):e0243629.
61. International Organization for Migration [Internet]. Migration health in the sustainable development goals: 'Leave No One Behind' in an increasingly mobile society. IOM Migration Health Division Position Paper, 2022. International Organization for Migration [cited 2022 August 23]. Available from: https://www.iom.int/sites/g/files/tmzbdl486/files/our_work/DMM/Migration-Health/mhd_position_paper_sdgs_02.09.2020_en.pdf
62. Scalabrini Centre of Cape Town [Internet]. Migrant and refugee access to public healthcare in South Africa. Scalabrini Centre of Cape Town and Sonke Gender Justice and Lawyers for Human Rights, 2019. Scalabrini Centre of Cape Town [cited 2022 September 20]. Available from: https://www.scalabrini.org.za/migrant-and-refuge e-access-to-public-healthcare-in-south-africa/
63. Southern African Development Community (SADC) [Internet]. Migration data in the Southern African Development Community (SADC). IOM UN Migration and Global Migration Data Portal Co-funded by

the European Union and the Government of Switzerland. Southern African Development Community (SADC), 2021 [cited 2022 September 20]. Available from: https://www.migrationdataportal.org/regional-data-overview/southern-africa
64. Rechel B, Mladovsky P, Ingleby D, Mackenbach JP, McKee M. Migration and health in an increasingly diverse Europe. The Lancet, 2013 April; 381 (9873): 1235-1245.
65. Dauda S, Imoro RJ. Ghana and COVID-19: Perspectives on livelihoods, health and living conditions of internal migrants in Accra. Cogent Social Sciences, 2022 May; 8 (1): 2072060.
66. Rédaction Africannews [Internet]. South Africa Advocacy group defends migrants' right of access to public healthcare. Rédaction Africannews, 2022 [cited 2022 September 20]. Available from: https://www.africannews.come/2022/08/05/south-africa-advocacy-group-defends-migrants-right-of-access-to-public-healthcare/
67. Vanyoro K. [Internet]. Migrants in South Africa have access to healthcare: why it's kicking up a storm? The Conversation Africa, 2022. The Conversation Africa, Inc [cited 2022 July 15]. Available from: https://www. theconversation.com/migrants-in-south-africa-have-access-to-healthcare-why-its-kicking-up-a-storm-1895 74
68. Zenda C. [Internet]. Are foreigners truly burdening South Africa's healthcare system? The Green Web Foundation. The Green Web Foundation, 2022 [cited 2022 September 20]. Available from: https://www. fairplanet.org/story/are-foreignerstruly-burdening-south-africas-healthcare-system
69. Sonke Gender Justice [Internet]. What does the law say about migrants and refugees accessing healthcare in South Africa? Sonke Gender Justice, 2021 [cited 2022 September 10]. Available from: https:// www.//genderjustice.org.za/card/refugees-migrants-and-health-care-in-south-africa-explained/what-does-the-law-say-about-migrants-and–refugees-accessing-healthcare-in-south-africa/
70. Ahmed S, Shommu NS, Rumana N, Gary Barron RS, Wicklum S, Turin TC. Barriers to access of primary healthcare by immigrant populations in Canada: A Literature Review. J Immigr Minor Health, 2016 Dec; 18 (6): 1522-1540.
71. Winters M, Rechel B, de Jong L, Pavlova M. A systematic review on the use of healthcare services by undocumented migrants in Europe. BMC Health Serv Res. 2018 Jan; 18 (1): 30.
72. Allegri C, Banks H, Devillanova C. Avoidable hospitalizations and access to primary care: comparisons among Italians, resident immigrants and undocumented immigrants in administrative hospital discharge records. EClinicalMedicine, 2022 March; 46: 101345.
73. Lurie MN, Williams BG, Zuma K, Mkaya-Mwamburi D, Garnett G, Sturm AW, Sweat MD, Gittelsohn J, Abdool Karim SS. The impact of migration on HIV-1 transmission in South Africa: a study of migrant and non-immigrant men and their partners. Sex Transm Dis. 2003 Feb; 30 (2): 149-156.
74. Ross J, Cunningham CO, Hanna DB. HIV outcomes among migrants from low- and middle-income countries living in high-income countries: a review of recent evidence. Curr Opin Infect Dis. 2018 Feb; 31 (1): 25-32.
75. Suphanchaimat R, Kantamaturapoj K, Putthasri W, Prakongsai P. Challenges in the provision of healthcare services for migrants: a systematic review through providers' lens. BMC Health Serv Res, 2015 Sept; 15: 390.
76. Gonah L, Corwin A, January J, Shamu S, Nyati-Jokomo Z, van der Putten M. Barriers to healthcare access and coping mechanisms among sub-Saharan African migrants living in Bangkok, Thailand: A qualitative study. Medical Journal of Zambia 2016 Feb; 43 (4): 238-246.
77. World Health African Region [Internet]. Health of refugees and migrants: regional situation analysis, practices, experiences, lessons learned and ways forward. World Health Organization, Africa, 2018 [cited 2022 September 10]. Available from: https://www.who.int/publications/i/item/health-of-refugees-and-migrants---who-european-region-(2018)
78. Webb V. Language in South Africa: the role of language in national transformation, reconstruction and development. Impact: Studies in language and society, 2002 August; 14: 78.
79. Louwrens K, Kotzé LJ, Ingeborg M, Albert E. Northern Sotho. München: Lincom, 1995 Jan; Pp. 4-11.
80. Rakgogo TJ, van Huyssteen L. A constitutional language name, lost in translation and its impact on the identity of the first language speakers. South African Journal of African Languages, 2019 July; 39 (2): 165-174.
81. Carter PM. Languages in the world: How history, culture, and politics shape language. Wiley, 2016 Jan. p. 139.
82. Ayorinde OA, Stefatos A, Miller S, Amanda R, Pallavi N, She J, Alghofaily A, Mngoma N. The salience and symbolism of number across cultural beliefs and practice. International Review of Psychiatry 2020 Feb-Mar; 33 (1-2): 179-188.

83. Riza E, Karnaki P, Gil-Salmerón A, Zota K, Ho, M, Petropoulou M, Katsas K, Garcés-Ferrer J, Linos A. Determinants of refugee and migrant health status in 10 European Countries: the Mig-healthcare project. International Journal of Environmental Research and Public Health, 2020 Aug; 17 (17): 6353.
84. Ridde V, Aho J, Ndao EM, Benoit M, Hanley J, Lagrange S, Fillol A, Raynault M-F, Cloos P. Unmet healthcare needs among migrants without medical insurance in Montreal, Canada. Global Public Health, 2020 Nov; 15:11, 1603-1616.
85. Odhiambo AJ, Forman L, Nelson LE, O'Campo P, Grace D. Legislatively Excluded, Medically Uninsured and Structurally Violated: The Social Organization of HIV Healthcare for African, Caribbean and Black Immigrants with Precarious Immigration Status in Toronto, Canada. Qualitative Health Research, 2022 April; 32(5):847-865.
86. Ventura E. [Internet]. TB and Migration in Southern Africa. International Organization for Migration (IOM). International Organization for Migration (IOM), UN Migration, 2016 March [cited 2022 September 22]. Available from: https://www.weblog.iom. Int/tb-and-migration-southern-africa
87. Faturiyele I, Karletsos D, Ntene-Sealiete K, Musekiwa A, Khabo M, Mariti M, Mahasha P, Xulu T, Pisa PT on behalf of EQUIP Innovation for Health Team. Access to HIV care and treatment for migrants between Lesotho and South Africa: a mixed methods study. BMC Public Health, 2018 May; 18, 668.
88. Steenberg, B. Structural vulnerabilities and healthcare services integration: HIV-positive Mozambican migrants in Johannesburg. Medicine Anthropology Theory | an open-access Journal in the Anthropology of Health Illness and Medicine, 2020 April; 7: 24-43.
89. Yorlets RR, Lurie MN, Ginsburg C, Hogan JW, Joyce NR, Harawa S, Collinson MA, Gómez-Olivé FX, White MJ. Validity of Self-Report for Ascertaining HIV Status among Circular Migrants and Permanent Residents in South Africa: A Cross-Sectional, Population-Based Analysis. *AIDS Behav*ior April, 2022; 29.
90. Rich C, Mavhu W, France NF, Munatsi V, Byrne E, Willis N, Nolan A. Exploring the beliefs, experiences and impacts of HIV-related self-stigma amongst adolescents and young adults living with HIV in Harare, Zimbabwe: A qualitative study. PLoS ONE, 2022 May; 17 (5): e0268498.
91. Stevenson S. [Internet] 2019. Are foreigners really entitled to free healthcare in South Africa? BHEKISISA Centre for Health Journalism, 2019 [cited 2022 September 22]. Available from: https://www.bheki sisa.org/article/2019-03-06-can-foreigners-get-medical-aid-free-healthcare-in-south-africa/
92. Mehlwana L. [Internet]. COVID-19: Foreign nationals speak out on unfair health treatment. Daily Maverick, 2021 [cited 2022 September 19]. Available from: https://www.dailymaverick.co.za/author/luvuyo-mehlwana/

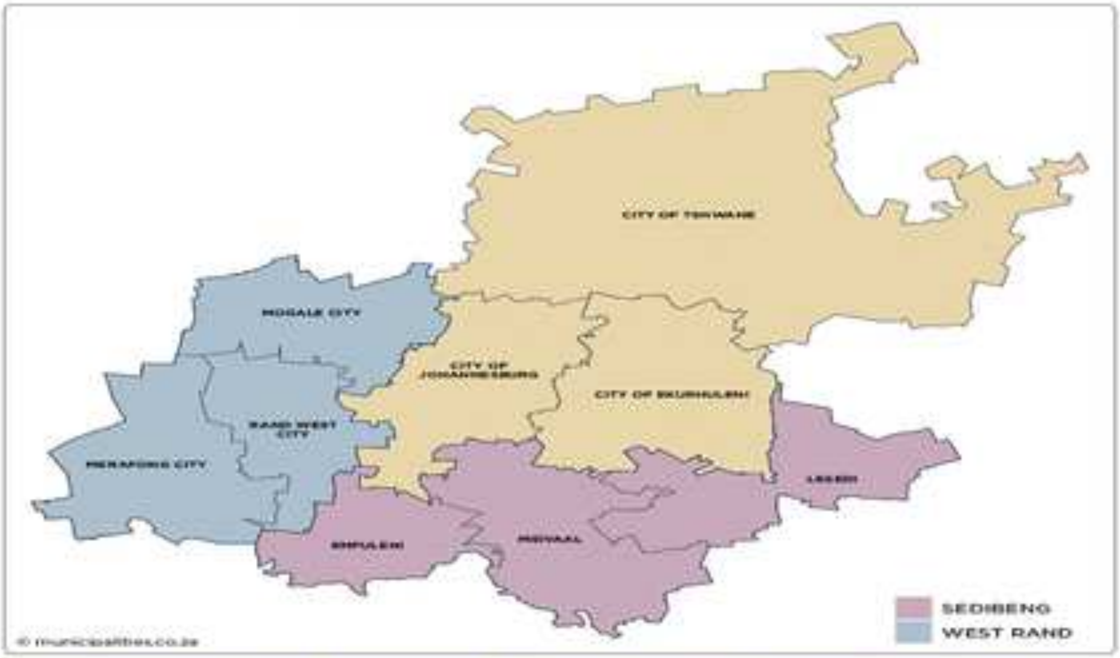

Fig. 1. Gauteng Province showing the metropolitan areas, which are Johannesburg, Tshwane, and Ekurhuleni, as well as the two districts municipalities of Sedibeng and West Rand, with their component city and local municipalities

Figure

Click here to access/download;Figure;Fig 2.tif

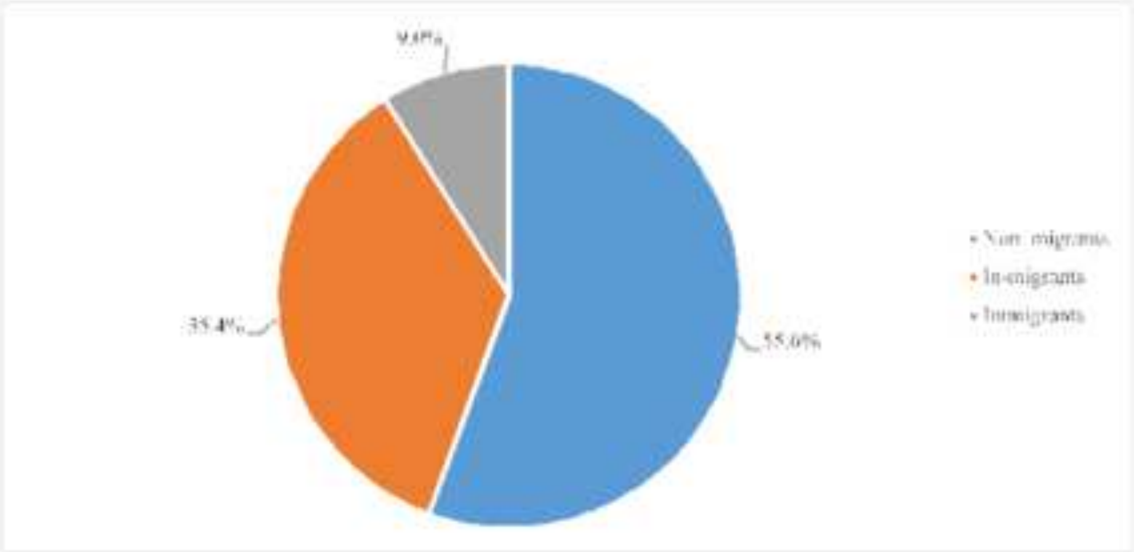

Fig. 1. Pie chart showing the proportion of non-migrants and migrants in Gauteng Province, South Africa, 2017- 2018 (N=4 872); Source: GCRO 2017/2018

Figure

Click here to access/download;Figure;Fig 3.tif

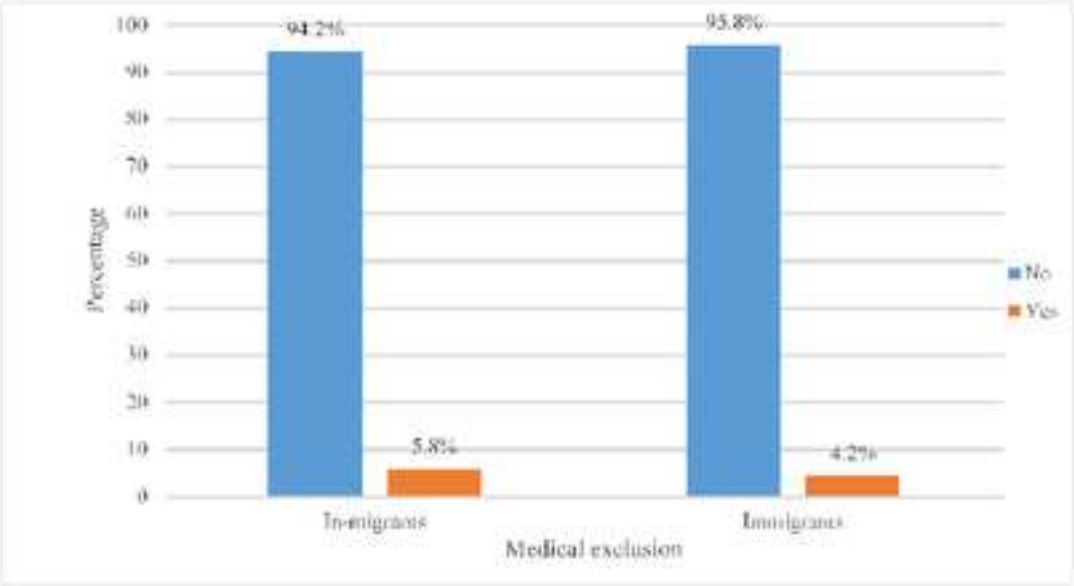

Fig 3. Bar chart showing the prevalence of medical exclusion among in-migrants and immigrants in Gauteng, South Africa, 2017-2018 (N=2162); Source: GCRO 2017-2018

 Click here to access/download;Figure;Fig 4.tif

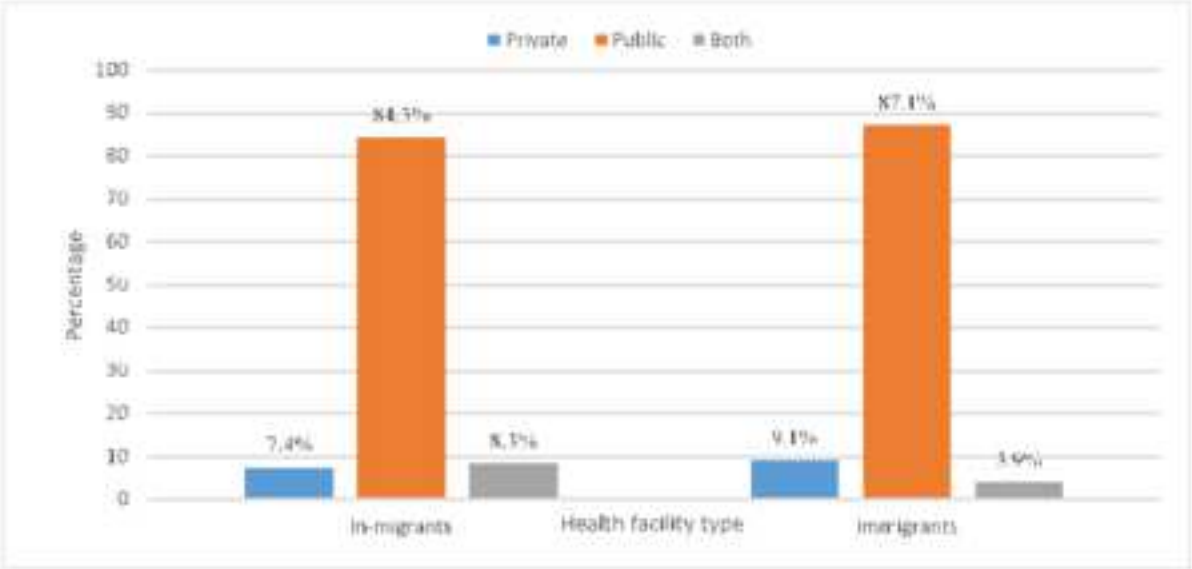

Fig. 4: Bar chart showing percentage distribution of health facility types utilized by migrants in Gauteng Province of South Africa, 2017- 2018 (N=2162); Source: GCRO 2017- 2018

 Click here to access/download;Figure;Fig 5.tif

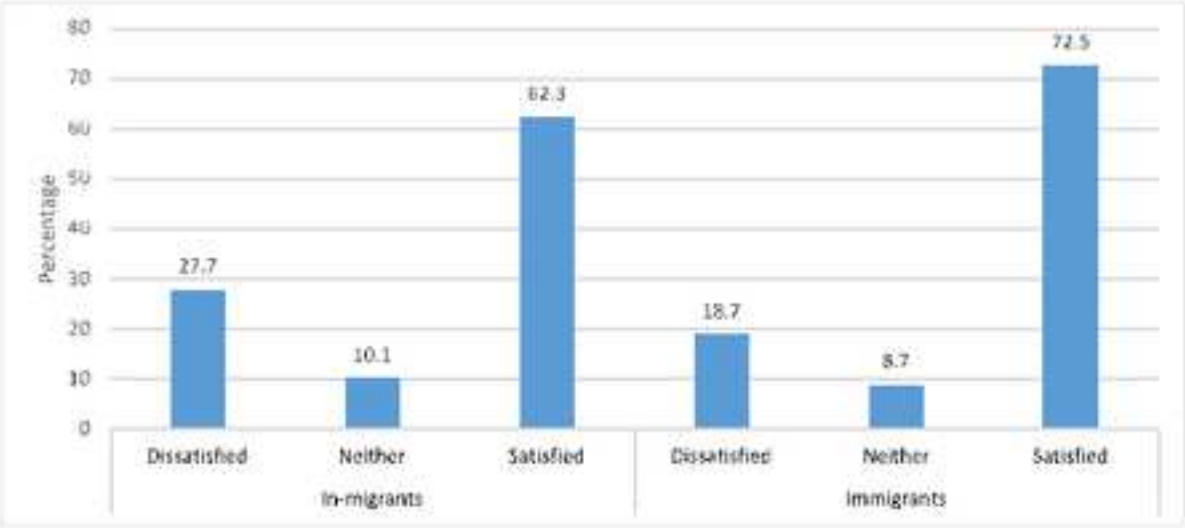

Fig. 5: Bar chart showing percentage distribution of health service satisfaction by migrants in Gauteng Province of South Africa, 2017-2018 (N=2162); Source: GCRO 2017-2018

Click here to access/download
Supporting Information
Final Do file_Edited 28_06_2022.do.docx

Data Review URL

Click here to download Data Review URL http://www.gcro.ac.za
