# Supplementary material for: Health services satisfaction and medical exclusion among migrant youths in Gauteng Province of South Africa: A cross-sectional analysis of the GCRO survey (2017−2018)
Source: PLoS One. 2023 Nov 29;18(11):e0293958. doi: 10.1371/journal.pone.0293958 (PMC10686501; doi:10.1371/journal.pone.0293958)
Supplement: Attachment — Submitted filename: Review comments_12.01.2023.docx [file pone.0293958.s002.docx]

**Health Services Satisfaction and Medical Exclusion among Migrant Youths in Gauteng Province of South Africa: A Cross-sectional Study of the GCRO Survey (2017−2018)**

**Title**

- The title is clear and precise.

**Abstract**

-In the abstract and the introduction, the authors summarized the main research question and key findings.

-There is no mention of health services satisfaction in the aim.

This study aimed to determine the prevalence and factors contributing to medical exclusion among migrant youths in Gauteng Province, South Africa.

-Please consider shortening your sentences. The second sentence under the method subtitle is very long.

-To reduce the word count the authors can just mention ρ≤0.05 (it covers the other two specified p-values).

“At the bivariate level, demographic (age, sex, and population group), economic (employed and any income) and health-related (no medical aid and household member with mental health) factors were significantly associated with medical exclusion (ρ≤0.05, ρ≤0.01 ρ≤0.001)”.

-Please consider paraphrasing the sentence below, it doesn’t read well.

“The adjusted odds ratio (AOR) of logistic regression indicated factors such as female gender (AOR 1.07, 95% CI 0.678, 1.705), no medical aid cover (AOR 1.23, 95% CI 0.450‒3.362), and neither (AOR 1.59, 95% CI 0.606‒4.174) or dissatisfied (AOR 4.29, 95% CI 2.528‒7.270) with the health services were predictors of medical exclusion”.

**Introduction**

-Please provide some migration statistics on what is happening currently in South Africa to give a clear picture of what is currently happening.

-Please elaborate, give examples of these factors, where were these studies conducted?

Factors associated with migrants’ exclusion from healthcare access have been cited in several studies [20-22].

-Please cite these studies

Also, other studies have mentioned medical xenophobia as one of the major barriers that migrants are faced with and this has been a hindrance to healthcare accessibility in South Africa.

**Methods**

-This sentence can be moved to the introduction.

In addition, SA Statistics (2020) reported an estimate of 2.9 million migrants who are presently residing in South Africa at mid-year 2020 [7].

-Avoid repetition

-Describe the study design of the current study (not the GCRO)

-It is not very clear how the authors got the sample size or how the potential recall bias was eliminated. A flow diagram on how the sample size was obtained would have been helpful.

However, the data analyzed in this study were limited to a total of 2,162 immigrants and in-migrants in order to eliminate any potential recall bias

**Results**

-For tables that do not fit in one page enable repeat table titles.

-Table 1, this is the first time the authors are mentioning a sample size of N=4 872, it is not clear how they arrived at this number.

-It would be great to stay consistent and use N for total number of participants instead of F as indicated in Table 1.

-All figures were blurry increasing the resolution of the figures to 600-1000dpi could help solve this problem.

-Page 7 “Bivariate analysis of factors associated with medical exclusion among in-migrants and immigrants” please consider rephrasing the paragraph.

- For Table 2 please consider labelling the chi2 column P-value and not ꭓ2.

-Table 3, it would be great to replace odds label as odds ratio since that is what the authors are presenting.

-Table 3 please present one overall p-value for the categorical variables.

**Discussion**

-Consider restructuring the discussion and organizing the paragraphs for easy flow of ideas. Please avoid introducing new ideas and concepts. Highlight your findings and substantiate your findings with available literature.

-Please discuss variables that were statistically significant in the multivariate analysis. Language was not statistically significant.

“Migrants who speak Sesotho, Sepedi and other languages (Other languages are Afrikaans, Swati, Tsonga, Tswana, Venda, Ndebele, Xhosa and English) as their household main languages were more likely to be excluded from health care services compared to those who speak IsiZulu as their household main language”

**Conclusion**

-It is clear and linked to the aim.

**Ethics**

-It is not clear whether this study was cleared by any ethical review committee.
