# Supplementary material for: Health services satisfaction and medical exclusion among migrant youths in Gauteng Province of South Africa: A cross-sectional analysis of the GCRO survey (2017−2018)
Source: PLoS One. 2023 Nov 29;18(11):e0293958. doi: 10.1371/journal.pone.0293958 (PMC10686501; doi:10.1371/journal.pone.0293958)
Supplement: Attachment — Submitted filename: Response to Reviewers.docx [file pone.0293958.s004.docx]

**PONE-D-22-33773R1**

**Health Services Satisfaction and Medical Exclusion among Migrant Youths in Gauteng Province of South Africa: A Cross-sectional Analysis of the GCRO Survey (2017−2018)**

**PLOS ONE**

**Editor Comments:**

Please include the following items when submitting your revised manuscript:

- A rebuttal letter that responds to each point raised by the academic editor and reviewer(s). You should upload this letter as a separate file labeled 'Response to Reviewers'.

**Authors response:**

A rebuttal letter showing each points raised by the academic editor and reviewer(s) have been made.

- A marked-up copy of your manuscript that highlights changes made to the original version. You should upload this as a separate file labeled 'Revised Manuscript with Track Changes'.

**Authors response:**

A marked-up copy of my manuscript that highlights the changes made to the original version have been made.

- An unmarked version of your revised paper without tracked changes. You should upload this as a separate file labeled 'Manuscript'.

**Authors response:**

An unmarked version of my revised paper without tracked changes have been made and is uploaded as a separate file labeled ‘Manuscript’.

**Journal Requirements:**
Please review your reference list to ensure that it is complete and correct. If you have cited papers that have been retracted, please include the rationale for doing so in the manuscript text, or remove these references and replace them with relevant current references. Any changes to the reference list should be mentioned in the rebuttal letter that accompanies your revised manuscript. If you need to cite a retracted article, indicate the article’s retracted status in the References list and also include a citation and full reference for the retraction notice.

**Authors response:**

References listed in this manuscript are complete and correct and there was no cited retracted papers. All relevant current references were cited in this manuscript.

**Additional Editor Comments:**

Authors are advised to effect changes in the main manuscript and not only in the response letter. To this end kindly submit a clean version and a tracked changes version.

**Authors response:**

We have effected all changes in the main manuscript in red colour ink and we are submitting a clean version and a tracked changes version.

**Reviewers’ Comments**

1. If the authors have adequately addressed your comments raised in a previous round of review and you feel that this manuscript is now acceptable for publication, you may indicate that here to bypass the “Comments to the Author” section, enter your conflict-of-interest statement in the “Confidential to Editor” section, and submit your "Accept" recommendation.

Reviewer #3: All comments have been addressed

Reviewer #4: (No Response)

 2. Is the manuscript technically sound, and do the data support the conclusions?

The manuscript must describe a technically sound piece of scientific research with data that supports the conclusions. Experiments must have been conducted rigorously, with appropriate controls, replication, and sample sizes. The conclusions must be drawn appropriately based on the data presented.

Reviewer #3: Yes

Reviewer #4: Yes

 3. Has the statistical analysis been performed appropriately and rigorously?

Reviewer #3: Yes

Reviewer #4: No

 4. Have the authors made all data underlying the findings in their manuscript fully available?

The [PLOS Data policy](http://www.plosone.org/static/policies.action#sharing) requires authors to make all data underlying the findings described in their manuscript fully available without restriction, with rare exception (please refer to the Data Availability Statement in the manuscript PDF file). The data should be provided as part of the manuscript or its supporting information, or deposited to a public repository. For example, in addition to summary statistics, the data points behind means, medians and variance measures should be available. If there are restrictions on publicly sharing data—e.g., participant privacy or use of data from a third party—those must be specified.

Reviewer #3: No

Reviewer #4: No

 5. Is the manuscript presented in an intelligible fashion and written in standard English?

PLOS ONE does not copyedit accepted manuscripts, so the language in submitted articles must be clear, correct, and unambiguous. Any typographical or grammatical errors should be corrected at revision, so please note any specific errors here.

Reviewer #3: No

Reviewer #4: Yes

 6. **Review Comments to the Author**

Please use the space provided to explain your answers to the questions above. You may also include additional comments for the author, including concerns about dual publication, research ethics, or publication ethics. (Please upload your review as an attachment if it exceeds 20,000 characters)

**Reviewer #3: Abstract:**

The abstract provides a comprehensive overview of the study's key findings and its relevance. However, it's important to include specific quantitative results or effect sizes to give readers a sense of the magnitude of the findings.

**Authors response:**

Thank you very much for your comments. Specific quantitative results/effects sizes of the study were included in the ‘Result Section’ of the Abstract. The specific results included were at the level of the univariate, bivariate and multivariate, as specified in the result section [See Page 0 of the Abstract section in Red colour].

**Introduction:**

In the introduction, consider adding a concise statement of the research objectives or hypotheses. This will help readers understand the specific questions the study aims to address.

**Authors response:**

The main and specific objective(s) stated below have been inserted in the main manuscript in red colour ink.

**Main Objective:**

The primary aim of this study was to document the health services satisfaction and medical exclusion among migrant youths in order to potentially inform future decisions for policy interventions.

**Specific objective:**

The specific objectives of this study are to: (1) describe the socio-demographics, economic and health-related characteristics by migration status; (2) to determine the prevalence of medical exclusion and health service satisfaction according to migration status; (3) to examine the factors associated with medical exclusion by migration status; and (4) to examine the predictors of medical exclusion among migrants in Gauteng Province of South Africa. [See Page 3 in Red]

Provide a brief rationale for why this study is important. Why is understanding health services satisfaction and medical exclusion among migrant youths in South Africa significant?

**Authors response:** The rationale and the significance below has been inserted in the main manuscript in red colour ink.

Using a nationally representative datasets allowed the authors to obtain a representative view on migrants’ perspectives on the satisfaction of health services and; and to investigate the relationships between demographics and medical exclusion practices. Therefore, the rationale for this study is its contribution to an emerging literature that examines health service satisfaction and medical exclusion among migrant youths in South Africa. Hence, its significance, the findings from this study will help to redesign the existing practical interventions that addresses the unmet health needs of youth migrants in South Africa. [See Page 3 in red colour]

**Methods:**
Include more details on the GCRO survey (e.g., sample size, data collection methods, survey instruments) to help readers understand the data source better.

**Authors response:**

**e.g.,**

**Sample size** - The Fig 1 below shows the diagram illustrating the stages carried out in the sample size selection of in-migrant and immigrant respondents. A two-year cohort of the GCRO study consisted of 24,889 respondents, out of which 4,872 respondents were sampled according to their assigned categories such as non-migrants, in-migrants, and immigrants. A total of 2,162 respondents involving in-migrants and immigrants were then sampled and utilized as the sample size for this study. In this study, the population were migrants aged 18‒29 years, stratified by 1,725 in-migrants and 437 immigrants, totalling 2,162 (See Figure 1). [See Page 5 in red ink colour].

**Data collection methods** – The GCRO is a nationally representative data in South Africa, and the survey methods employed were included in the main manuscript in red ink colour. However, any information you need to have can be accessed via the GCRO website (https://www.gcro. ac.za) [See Page 4 and Page 5, respectively].

**Survey instruments –** The survey instruments used were questionnaire, as the GCRO is a quantitative data.

When discussing statistical analysis, specify the exact statistical tests or modeling techniques used for various analyses.

**Authors response:**

The statistical tests of the binary logistic regression, has the dependent variable, which is a dichotomous (binary) variable, coded as 0 or 1. It specifically helps to determine how much a dependent variable (Y) is affected by one or more independent variables (X), where Y is the dependent variable, X is the independent (explanatory) variable, B is the slope and a is the intercept as well as Ɛ is the residual (error). However, the binary regression model is expressed in terms of the logit instead of $\gamma:logit = \mathbb{L}\mathfrak{i =}\beta o + \betaıXı +\cdot\cdot\cdot+ \beta\kappa X\kappa\cdot$[See Page 6 and Page 7, inserted in the main manuscript in red colour]

**However,**

**Univariate analysis** explores each variable in a data set, separately. It looks at the range of values, as well as the central tendency of the values. It describes the pattern of response to the variable. It describes each variable on its own. Descriptive statistics describe and summarize data. There are three common methods for performing univariate analysis: (1) Summary Statistics (measures of central tendency and dispersion measures) [Summary statistics information have been included in Table 1 – Page 7 to Page 8], (2) Frequency Distributions, and (3) Charts. [The univariate analysis were used to express these sub-section in the main manuscript such as prevalence of medical exclusion according to migration status, Percentage distribution of the proportion of migrants and the health facility type utilized, and Percentage distribution of migrants and health service satisfaction] [See Page 6 and Page 8 in the main manuscript in red colour ink]

**Bivariate analysis** is stated to be an analysis of any concurrent relation between two variables or attributes. This study explores the relationship of two variables as well as the depth of this relationship to figure out if there are any discrepancies between two variables and any causes of this difference. The types of bivariate data analysis are the: Numerical and Numerical (in this type, both the variables of bivariate data, independent and dependent, are having numerical values) and Categorical and Categorical (when both the variables are categorical).

For a bivariate or simple regression with an independent variable x and a dependent variable y, the equation is: y=bx+a, where  y is the dependent variable, x is the independent variable, a is the point where the line of best fit intersects the y-axis and b is the angle of the line. [See Page 6 in red colour ink, inserted in the main manuscript in red colour].

Mention how missing data were handled, if applicable.

**Authors response:** The missing data were found on certain variables where the totals are unequal, so such variables were dropped during the analysis.

**Results:**
In the results section, consider breaking down the presentation of findings into subsections to make it easier for readers to navigate. For example, you could have subsections for demographics, health facility usage, satisfaction, and exclusion.

**Authors response:** The presentation findings were broken down into sub-sections for readers to navigate. From Page 7 to Page 9, you will see the sub-sections in bold italics as follows:

-Socio-demographic Characteristics [See Page 7]

-Prevalence of medical exclusion according to migration status [See Page 8]

-Percentage distribution of the proportion of migrants and the health facility type utilized [See Page 8]

-Percentage distribution of migrants and health service satisfaction [See Page 8]

-Bivariate analysis of factors associated with medical exclusion [See Page 8]

-Multivariate analysis of the unadjusted and adjusted logistic regression of the factors associated with medical exclusion [See Page 9]

When presenting prevalence percentages, consider providing 95% confidence intervals, especially when comparing different groups. This adds precision to the estimates.

**Authors response:** This has been inserted accordingly in Red colour ink.

-Prevalence of medical exclusion according to migration status [See Page 8]

-Percentage distribution of the proportion of migrants and the health facility type utilized [See Page 8]

-Percentage distribution of migrants and health service satisfaction [See Page 8]

For significant findings, briefly discuss their practical implications. What do these results mean for healthcare policies or interventions targeting migrant youths?

**Authors response:** Practical implications of the study findings for healthcare policies or interventions targeting migrant youths were included in the sub-section: “Implications of findings on medical exclusion of migrants in South Africa**”**. [See Page 13].

**Discussion:**

The discussion section should go beyond summarizing the results. It's an opportunity to interpret the findings in the context of existing literature. How do the study's results align with or diverge from prior research on this topic?

**Authors response:** The discussion section did not just involve summarizing the results, but it interpreted the findings in the context of existing literature. Summary of results provide the paragraphs and the flow of the findings to align with the context of existing studies. [See Page 11 to Page 13]

Consider discussing potential explanations for any unexpected or counterintuitive results. Were there limitations in the study design that could account for these findings?

**Authors response:** Thank you very much for your comments. However, discussions on the potential explanation for the results were included in the sub-section: “Strengths and limitations”. [See Page 14]

Highlight the policy implications of the findings. If possible, suggest specific policy changes or interventions that could address the issues identified in the study.

**Authors response:** Thank you very much for your comments. However, policy implications of the findings were included in the “Implications of findings on medical exclusion of migrants in South Africa**”**. [See Page 13].

**Limitations:**

The limitations section is essential. However, it could be more detailed. Mention any potential sources of bias or limitations in the data source (e.g., self-reporting bias, sampling limitations) that could have influenced the results.

**Authors response:** Limitations of the study is the common methodological limitations of studies. We included all the limitations that are applicable to this study in the sub-section “Strengths and Limitations” [See Page 14].

**Conclusion:**

Summarize the key takeaways of the study concisely. Restate the main findings and their implications.

**Authors response:** Thank you very much for your comments. The conclusion is intended to help the reader understand why the research should matter to the readers after they have finished reading the paper, and a conclusion is not merely a summary of points or a re-statement of the research problem but a synthesis of key points. But the significant findings were captured on the conclusion section [See Page 14].

**Recommendation:**
If there are specific recommendations based on the findings, provide them here. For example, you could recommend changes in healthcare policies or interventions to improve the healthcare experiences of migrant youths.

**Authors response:** Thank you very much for your comments. However, specific recommendations in this study were grounded on the findings of the study, where recommendations were specifically stated in the “Recommendation section”. [See Page 14].

**Acknowledgments:**
Ensure that all individuals, institutions, or organizations deserving acknowledgment are included in this section, and consider specifying their contributions if relevant.

**Authors response:** Thank you very much for your comments. All-important individuals and institutions were full acknowledged in this study [See Page 15].

Remember to maintain a clear and logical flow between sections and subsections throughout the paper. These comments should help improve the clarity, detail, and overall quality of the paper.

**Authors response:** Thank you very much for your comments. This manuscript was sent to a Professional Editor to edit it for a clear and logical flow throughout the paper between sections and sub-sections.

**Reviewer #4:**

♣ Authors should include a brief description about how the weighting was implemented for this analysis.

**Authors response:**  A brief description of weighting was inserted in the main manuscript. [See Page 7 in Red colour ink].

♣ Authors should include more information about the collinearity assessment in the manuscript.

**Authors response:** Multicollinearity assessment was carried out with STATA statistical software. It is documented in the main manuscript [See Page 7 in Red colour ink].

♣ I think that the proportions of those who were medically excluded should be included in the descriptive analysis.

**Authors response:** The proportions of those who were medically excluded have been included in the descriptive analysis (Table 2) [See Page 9 to Page 10 in Red colour ink].

♣ Authors should include some definition of key terms in the introduction “medical exclusion” and others.

**Authors response:** Thank you very much for your comment. Medical exclusion was used in this study and the definition was mentioned in Page 5 of the manuscript. However, we only stated that some studies used the one word ‘medical xenophobia’ as our study used medical exclusion, and we defined what we meant as medical exclusion in this study. [Page 5]

♣ The way medical exclusion was conceptualized for this study has some limitation that I was expecting the authors to highlight. If a respondents had personal challenges that prevented him from seeking care, then it does not mean they have been excluded.

**Authors response:** The definition of medical exclusion used in this study was clearly defined in the sub-section “Variable Measurement” [See Page 5 in Red colour ink].

♣ Although others provided a link which seem to be a repository for the data used for this analysis it was not included in the main manuscript.

**Authors response:** Thank you for the comment, but as a result of ethical concerns the data cannot be shred or kept in the repository. We need to comply with the ethics framework not to share the data, however, we deposited the Do-files we created for the analysis as Appendices. [See Page 5 in Red colour ink].

♣ Also, I note that authors used secondary data for this analysis. It will be great for the authors to include ethics approval status for the original survey.

**Authors response:** Thank you for the comment, we have included the ethics approval and consent to participate in the main manuscript. [See Page 7].

♣ Generally, I see that the responses were done directly in the response documents but changes were not effected in the main manuscript.

**Authors response:** See all changes made in red colour ink in the main manuscript. [See Page 0 to Page 19].
